# Persistent Replication of a Chikungunya Virus Replicon in Human Cells Is Associated with Presence of Stable Cytoplasmic Granules Containing Nonstructural Protein 3

**DOI:** 10.1128/JVI.00477-18

**Published:** 2018-07-31

**Authors:** Roland Remenyi, Yanni Gao, Ruth E. Hughes, Alistair Curd, Carsten Zothner, Michelle Peckham, Andres Merits, Mark Harris

**Affiliations:** a School of Molecular and Cellular Biology, Faculty of Biological Sciences, University of Leeds, Leeds, United Kingdom; b Astbury Centre for Structural Molecular Biology, University of Leeds, Leeds, United Kingdom; c Institute of Technology, University of Tartu, Tartu, Estonia; Washington University School of Medicine

**Keywords:** Airyscan, confocal microscopy, neglected tropical diseases, nonstructural viral proteins, nucleopore, self-labeling tag, superresolution microscopy, video microscopy, virus-cell interaction

## Abstract

Chikungunya virus (CHIKV), a mosquito-borne human pathogen, causes a disabling disease characterized by severe joint pain that can persist for weeks, months, or even years in patients. The nonstructural protein 3 (nsP3) plays essential roles during acute infection, but little is known about the function of nsP3 during chronic disease. Here, we used subdiffraction multicolor microscopy for spatial and temporal analysis of CHIKV nsP3 within human cells that persistently replicate replicon RNA. Round cytoplasmic granules of various sizes (i) contained nsP3 and stress granule assembly factors 1 and 2 (G3BP1/2), (ii) were next to double-stranded RNA foci and nsP1-positive structures, and (iii) were close to the nuclear membrane and the nuclear pore complex protein Nup98. Analysis of protein turnover and mobility by live-cell microscopy revealed that the granules could persist for hours to days, accumulated newly synthesized protein, and moved through the cytoplasm at various speeds. The granules also had a static internal architecture and were stable in cell lysates. Refractory cells that had cleared the noncytotoxic replicon regained the ability to respond to arsenite-induced stress. In summary, nsP3 can form uniquely stable granular structures that persist long-term within the host cell. This continued presence of viral and cellular protein complexes has implications for the study of the pathogenic consequences of lingering CHIKV infection and the development of strategies to mitigate the burden of chronic musculoskeletal disease brought about by a medically important arthropod-borne virus (arbovirus).

**IMPORTANCE** Chikungunya virus (CHIKV) is a reemerging alphavirus transmitted by mosquitos and causes transient sickness but also chronic disease affecting muscles and joints. No approved vaccines or antivirals are available. Thus, a better understanding of the viral life cycle and the role of viral proteins can aid in identifying new therapeutic targets. Advances in microscopy and development of noncytotoxic replicons (A. Utt, P. K. Das, M. Varjak, V. Lulla, A. Lulla, A. Merits, J Virol 89:3145–3162, 2015, https://doi.org/10.1128/JVI.03213-14) have allowed researchers to study viral proteins within controlled laboratory environments over extended durations. Here we established human cells that stably replicate replicon RNA and express tagged nonstructural protein 3 (nsP3). The ability to track nsP3 within the host cell and during persistent replication can benefit fundamental research efforts to better understand long-term consequences of the persistence of viral protein complexes and thereby provide the foundation for new therapeutic targets to control CHIKV infection and treat chronic disease symptoms.

## Abstract (precis)

Chikungunya virus (CHIKV) is a reemerging alphavirus transmitted by mosquitos and causes transient sickness but also chronic disease affecting muscles and joints. No approved vaccines or antivirals are available. Thus, a better understanding of the viral life cycle and the role of viral proteins can aid in identifying new therapeutic targets. Advances in microscopy and development of noncytotoxic replicons (A. Utt, P. K. Das, M. Varjak, V. Lulla, A. Lulla, A. Merits, J Virol 89:3145–3162, 2015, https://doi.org/10.1128/JVI.03213-14) have allowed researchers to study viral proteins within controlled laboratory environments over extended durations. Here we established human cells that stably replicate replicon RNA and express tagged nonstructural protein 3 (nsP3). The ability to track nsP3 within the host cell and during persistent replication can benefit fundamental research efforts to better understand long-term consequences of the persistence of viral protein complexes and thereby provide the foundation for new therapeutic targets to control CHIKV infection and treat chronic disease symptoms.

## INTRODUCTION

Chikungunya virus (CHIKV), a reemerging arbovirus of the Alphavirus genus, causes a transient illness with debilitating symptoms (fever, headache, rash, myalgia, and arthralgia). Chronic disease is common, and joint pain can persist for months to years (1–3). Half of the patients from the recent Latin American outbreak may develop chronic inflammatory rheumatism, raising the health burden of musculoskeletal disease in areas of endemicity (4, 5). During acute infection, this cytotoxic virus induces apoptosis, leading to direct tissue injury and local inflammation (6–8). Biopsies have also revealed the persistence of CHIKV antigens and RNA in synovial macrophages and muscle tissue (1, 9). CHIKV also persists in mice and nonhuman primate models (10–13). Chronic disease may be a consequence of persistent, replicating, and transcriptionally active CHIKV RNA (13), but an understanding of CHIKV's long-term effect is still emerging.

**FIG 1:**
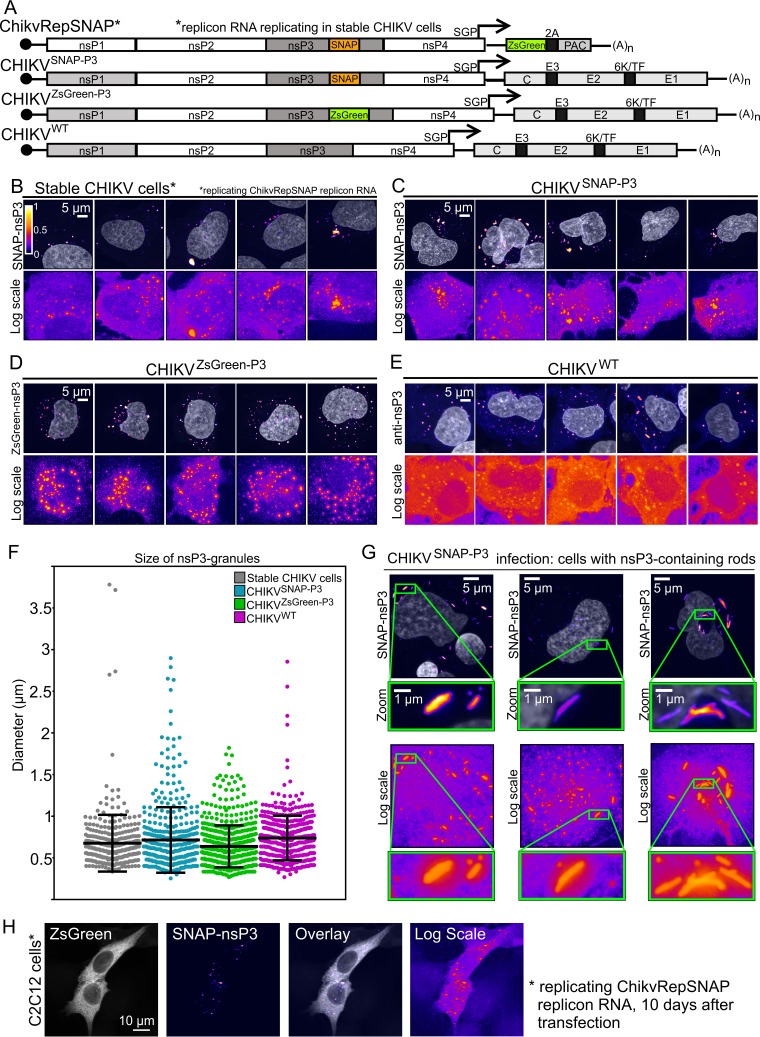
nsP3 has a granular distribution in stable CHIKV cells and infected HuH-7 cells. (A) Schematic representation of tagged reporter viruses and noncytotoxic replicon encoding SNAP-nsP3. SGP, subgenomic promoter; PAC, puromycin-*N*-acetyltransferase; 2A, foot-and-mouth disease virus (FMDV) 2A autoprotease. (B) Subdiffraction confocal microscopy of BG-647-SiR-labeled stable CHIKV cells, imaged in the far-red channel. Cells were chemically fixed and stained with fluorescent BG-647-SiR, which irreversibly binds SNAP-tagged proteins. (C to E) Naive HuH-7 cells were infected with viral stocks of CHIKV^SNAP-P3^, CHIKV^ZsGreen-P3^, or CHIKV^WT^. For cells infected with CHIKV^ZsGreen-P3^, images were acquired in the green channel. Cells infected with CHIKV^WT^ were immunostained with anti-nsP3 antibodies to visualize untagged nsP3. Data shown are maximum-intensity projections of Z-stacks acquired on an Airyscan confocal system, operated in the superresolution mode. To enhance the appearance of dim structures, Icy software (84) was used to pseudocolor image channels with the predefined Fire lookup table based on pixel intensity. Color bars indicate the relative range of pixel intensity (white = high, purple = low, from 0 arbitrary units to 1). Nuclear counterstain (gray) was overlaid as a reference. Images displayed in the Fire view, based on a logarithmic scale, illustrate both high-intensity and low-intensity granules in the same image. (F) Size of nsP3-containing granules. Diameters of granules were extracted from the maximum-intensity projections of a total of 10 fields of view (FOVs; including panels B to E and five additional FOVs). Feret diameters represent the maximum distance between any two points of the extracted surface. (G) Airyscan microscopy of rod-containing cells infected with CHIKV^SNAP-P3^. Zoomed-in views of nsP3-containing rods are provided in insets. (H) Mouse myoblast cell line (C2C12) replicating the SNAP-tagged noncytotoxic replicon. At 10 days after transfection and puromycin selection, cells were fixed and stained for SNAP-nsP3 with BG-TMR-Star. The ZsGreen channel is presented in grayscale view.

The ∼12-kb positive-sense RNA genome of CHIKV encodes four nonstructural proteins, nsP1 to nsP4, which make up the viral replication and transcription complex (Fig. 1A) (reviewed in reference 14). A subgenomic RNA expresses six structural proteins. Cellular responses to infection include apoptosis, interferon signaling, stress granule (SG) formation, unfolded protein response, host cell shutoff, and autophagy (reviewed in reference 15). Previous research on alphaviruses established the vital role that nsP3 plays in counteracting cellular responses (16–20) and identified essential protein-protein interactions between nsP3 and host proteins (16, 21–23). However, few studies have systematically investigated the long-term effect of persistently replicating CHIKV RNA and continued expression of proteins such as nsP3 on human cells. Although recent studies characterize the formation of organelles that contain nsP3 during acute infection and transient replication (16, 24–27), a corresponding characterization during persistent CHIKV replication is missing. To address these gaps, we sought to further develop CHIKV replicons capable of persistent replication in human cells and to harness this system for analysis by subdiffraction multicolor microscopy.

We previously characterized transient replication of CHIKV replicons in mammalian and invertebrate cell lines (27) and tagged nsP3 with the versatile SNAP tag for advanced fluorescence microscopy applications (26). The development of a noncytotoxic CHIKV replicon allowed the establishment of persistent replication in a human cell line (28). Here, we extended the SNAP-based labeling system to this noncytotoxic CHIKV replicon and generated a human cell line that persistently replicates replicon RNA and stably expresses SNAP-tagged nsP3. We then characterized nsP3-containing cytoplasmic granular organelles by subdiffraction multicolor microscopy. We used this technique to address questions relating to the subcellular localization of nsP3-containing granules and their stability, composition, and motility. This report is the first to shed light on the persistence of stable intracellular granules of nsP3 within human cells. In turn, understanding the link between the persistence of stable viral protein complexes and pathogenesis has relevance to future studies of chronic CHIKV disease.

(This article was submitted to an online preprint archive [29].)

## RESULTS

### Development of a stable human-origin cell line carrying a SNAP-tagged CHIKV replicon and superresolution microscopy of nsP3-G3BP-containing granules.

To determine the intracellular distribution of nsP3, we previously generated a SNAP-tagged replicon construct (26). Whereas this replicon is cytotoxic and replicates transiently, noncytotoxic replicons can establish persistent replication in the human cell line HuH-7 (28). To improve the HuH-7 CHIKV cell line, we added a SNAP-tagged nsP3 to a noncytotoxic replicon (Fig. 1A) and selected puromycin-resistant cells, which are called stable CHIKV cells throughout this paper. Silicon-rhodamine-conjugated O^6^-benzylguanine probes (BG-647-SiR) labeled SNAP-nsP3 and revealed nsP3-containing granules (Fig. 1B) comparable to those formed by a wild-type virus, CHIKV^WT^, and those formed by CHIKV^SNAP-P3^ and CHIKV^ZsGreen-P3^ viruses harboring SNAP- or ZsGreen-tagged nsP3 (Fig. 1C to E). Analysis of the sizes of nsP3-containing granules formed in stable CHIKV cells also showed size distributions and diameters comparable to those of granules formed during infection with tagged and untagged viruses (Fig. 1F). Further experiments focused on the characterization of these nsP3-containing granules. Whereas cells infected with CHIKV^ZsGreen-P3^ displayed only a granular nsP3-ZsGreen distribution pattern, cells infected with CHIKV^SNAP-P3^ also made rod-like structures (Fig. 1G), as described previously (26, 27). However, the presence of rods did not correlate with infectivity, as ZsGreen- and SNAP-tagged viruses replicated to similar titers (Table 1). Although the rest of this study used stable CHIKV cells derived from HuH-7 cells, the SNAP-tagged noncytotoxic replicon ChikvRepSNAP could also replicate in the C2C12 mouse myoblast cell line (Fig. 1H).

**TABLE 1:** Viral titers of CHIKV^SNAP-P3^ and CHIKV^ZsGreen-P3^

CHIKV construct	Virus titer (PFU/ml)^*a*^		
	24 h	48 h	72 h
CHIKV^SNAP-P3^	5.5 × 10^5^	4.4 × 10^7^	1.4 × 10^7^
CHIKV^ZsGreen-P3^	8.6 × 10^4^	6.1 × 10^7^	1.4 × 10^7^

a Inoculum obtained from supernatant at 24, 48, and 72 h after infection.

**FIG 2:**
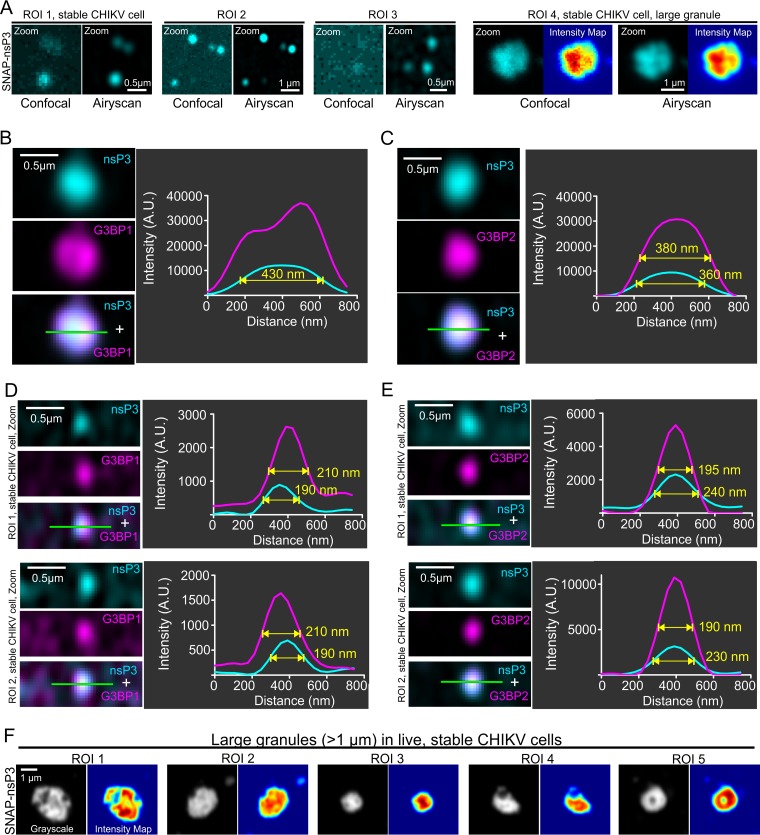
Characterization of nsP3-G3BP1/2 interaction by subdiffraction microscopy. (A) Comparison of confocal and Airyscan images. Airyscan provided improved resolution and signal-to-noise ratios (SNRs). Airyscan was able to image faint clusters that did not resolve well with standard confocal microscopy (ROI 1 to 3). Note that we use the term “granules” for these protein clusters. Airyscan also revealed differences in fluorescence intensity within large granules that were not apparent with confocal imaging (ROI 4). (B to E) SNAP-nsP3 (cyan) was stained with BG-647-SiR as described in the legend to Fig. 1. G3BP1 or G3BP2 (magenta) was immunostained with specific antibodies. Images were acquired with an Airyscan microscope operated in superresolution mode. For high-contrast display of nsP3-containing granules, contrast was optimized within each image by adjusting the view range in the histogram viewer window of Icy software. Overlaps between cyan and magenta layers appear in white (panels “nsP3 + G3BP1/2”). Line profiles that plot the fluorescence intensity along the line of interest (green) are provided as well, along with the full-width at half maximum (FWHM) of granules. Fluorescence intensity was measured in arbitrary units (A.U.). Images represent single slices, which were extracted from Z-stacks. (F) Live-cell Airyscan microscopy of large granules. Stable CHIKV cells were stained with BG-647-SiR and imaged with an Airyscan microscope operated in the Fast Airyscan mode. Fluorescence intensity maps were created in Icy software and represent relative pixel intensity according to the Jet color map.

CHIKV nsP3 sequesters G3BP1/2 when expressed alone (17), in the context of a replicon (16, 26), or during virus infection (24, 25), thereby interfering with SG responses. Recent subdiffraction microscopy revealed stable substructures of G3BP1 protein within SGs (30, 31). To determine whether nsP3-containing granules also sequestered G3BP1/2 proteins and contained similar substructures, we imaged stable CHIKV cells with Airyscan microscopy. Airyscan or image scanning microscopy (32, 33) relies on array detectors to reassign photon pixels and oversample the pattern from diffracted light, thereby improving image resolution (1.7-fold) and sensitivity (34). Airyscan outperformed standard confocal microscopy and was sensitive enough to detect small granular structures of nsP3 (Fig. 2A, region of interest [ROI] 1 to 3). Whereas nsP3 appeared to have a diffuse distribution in confocal images, the improved resolution of the Airyscan microscope uncovered an uneven distribution in a large (1.2-μm-diameter) granule, consistent with the presence of substructure (Fig. 2A, ROI 4). Both G3BP1 (Fig. 2B) and G3BP2 (Fig. 2C) were colocalized with these granules, which also had a high fluorescence intensity. The higher sensitivity of the Airyscan method also made small clusters of nsP3 more visible; these clusters had full width at half maximum (FWHM) of 190 to 240 nm and were about 10 times less intense (Fig. 2D and E, see line profiles of fluorescence intensity) than larger granules (Fig. 2B and C, FWHM of 360 to 430 nm). Lastly, live-cell imaging also confirmed that fluorescent nsP3 signals in large granules were nonuniformly distributed within each granule (Fig. 2F).

### Juxtaposition of nsP3-containing granules, dsRNA foci, nsP1-positive structures, nuclear membrane, and Nup98.

During the viral life cycle, nsP3-containing granules sequester G3BP1, thereby blocking SG assembly (16, 17). The relationship between cytoplasmic nsP3-G3BP1 complexes and CHIKV RNA synthesis is less clear; viral double-stranded RNA (dsRNA) foci overlap minimally with nsP3-G3BP1-positive clusters in replicons (16). Moreover, few dsRNA foci colocalized with G3BP2 puncta at 6 h postinfection, and even fewer overlapped after 8 h (25). Recently, it was reported that large cytoplasmic and small plasma-membrane-bound G3BP1-nsP3 complexes colocalize with viral genomic RNA during CHIKV infection, with dsRNA foci forming nearby (24). Structural and biochemical data also support a model in which a matrix of nsP3-G3BP1-containing complexes stabilizes replication complexes and shields viral replicative intermediates from the RNA degradation machinery or cytosolic dsRNA sensors (35). Thus, nsP3-G3BP1/2-containing granules appear to play roles in addition to sequestering SG-related proteins.

**FIG 3:**
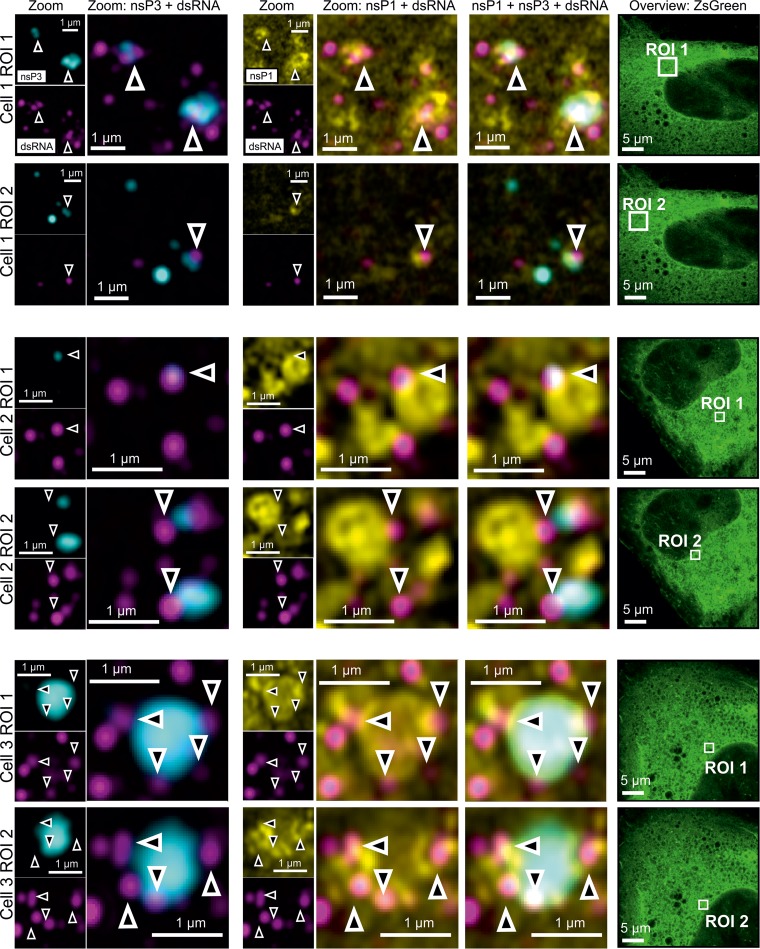
Four-color microscopy of nsP3, dsRNA, nsP1, and ZsGreen. Stable CHIKV cells were fixed and probed for nsP3 (cyan), dsRNA (magenta), nsP1 (nsP1), and ZsGreen (green) by a combination of SNAP tag labeling and indirect immunofluorescence assays. Images were taken with an Airyscan microscope operated in the superresolution mode. Overlay images are a combination of the nsP3, nsP1, and dsRNA layers as indicated. The zoomed-out ZsGreen channel is shown as a separate reference, with the corresponding ROI marked by a white box. Arrowheads indicate regions of proximity between nsP3, dsRNA, and nsP1.

To further explore the spatial relationship between nsP3 and replication sites in stable CHIKV cells, we visualized SNAP tag-labeled nsP3, together with immunostaining for nsP1 and dsRNA. The antibody against dsRNA was previously used to identify alphavirus replication complexes (36). The fluorescence of ZsGreen in stable CHIKV cells served as an indirect readout of the viral subgenomic RNA (Fig. 1A, cartoon). Rather than completely overlapping with larger nsP3-containing granules, dsRNA foci were in a proximal location and often juxtaposed (Fig. 3, arrowheads). In another example, a dsRNA focus coincided with a smaller nsP3-containing cluster (Fig. 3, cell 2, ROI 1, arrowhead). Ring-like structures coated with nsP1 were also near these dsRNA foci (Fig. 3). The proximity of dsRNA foci, nsP1-coated structures, and nsP3-containing granules suggested that nsP3-containing granules not only sequestered G3BP1/2 protein but also played a role in viral replication.

**FIG 4:**
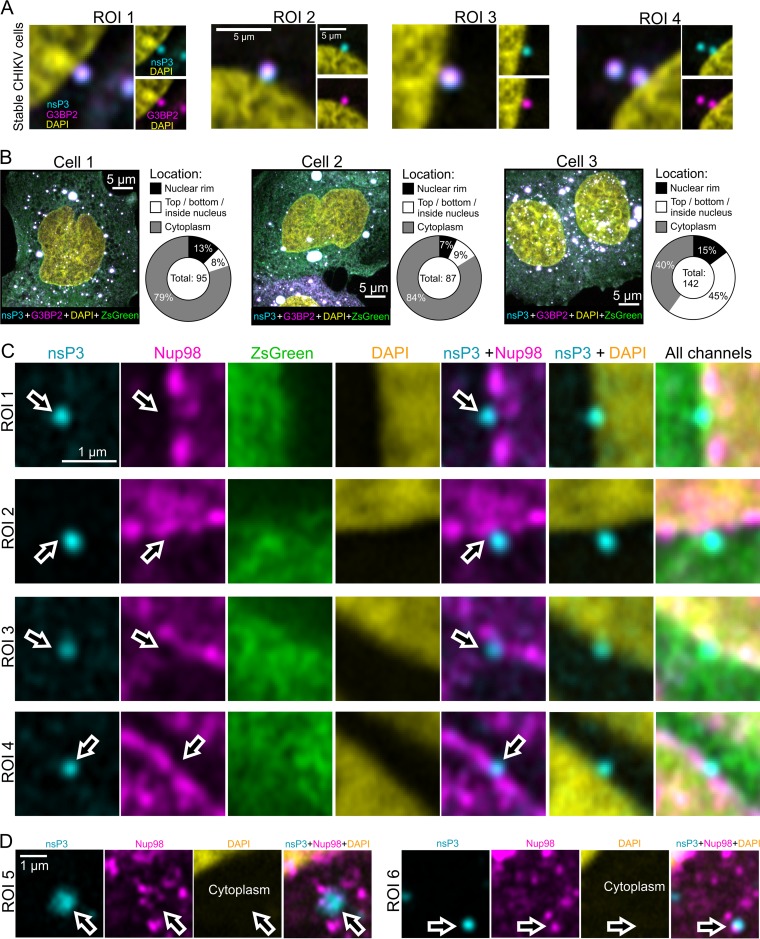
Association of nsP3-containing granules with nuclear membrane and Nup98. (A) Fixed, stable CHIKV cells were examined for the presence of nsP3 (cyan) and G3BP2 (magenta) at the nucleus (stained with DAPI; yellow). As shown in Fig. 2, both G3BP1 and G3BP2 colocalize with nsP3. Here we probed for G3BP2, since immunostaining had a higher SNR than G3BP1 staining. ROIs 1 to 4 are high-magnification views of granules associating with nuclei. (B) Quantification of association of nsP3-containing granules with nucleus. Z-stacks of three FOVs containing cells 1 to 3 were scored for the location of granules in three categories: (i) nuclear rim, (ii) top, bottom, or inside nucleus, and (iii) cytoplasm. (C) Fixed, stable CHIKV cells were examined for the presence of nsP3 (cyan) and Nup98 (magenta) at the nucleus (stained with DAPI; yellow). Arrows serve as digital fiducial markers and point toward regions where nsP3 is close to Nup98-positive cellular structures. Images are single slices extracted from Z-stacks that were taken with an Airyscan microscope operated in the superresolution mode. (D) Samples were prepared as described for panel C. Chosen ROIs are in perinuclear, cytoplasmic areas. Arrows point to nsP3-containing granules that are associated with cytoplasmic Nup98-positive structures.

As described above, nsP3-containing granules were part of a unique microenvironment that also housed dsRNA foci and nsP1. Moreover, a fraction of granules containing nsP3 and G3BP2 were located close to the nuclear membrane (Fig. 4A and B). The nuclear transport factor 2 (NTF2)-like domain of G3BP1 has previously been cocrystallized with FXFG (phenylalanine-glycine motif, where X is usually serine) nucleoporin (Nup) repeat peptides (37) and overexpressed G3BP1 interacts with some nucleoporins, whereas a mutant of G3BP lacking the binding site does not (38).

To further characterize the environment at the nuclear membrane of stable CHIKV cells, we probed for the nuclear pore complex protein Nup98. nsP3-containing granules were detected (i) at the nuclear membrane (Fig. 4C), flanked by Nup98-containing regions, and (ii) near cytoplasmic clusters of Nup98 (Fig. 4D). Thus, we were able to visualize a subset of nsP3-containing granules at the nuclear membrane and near Nup98-containing structures by Airyscan microscopy.

### Imaging the dynamics of nsP3-containing granules within stable CHIKV cells.

**FIG 5:**
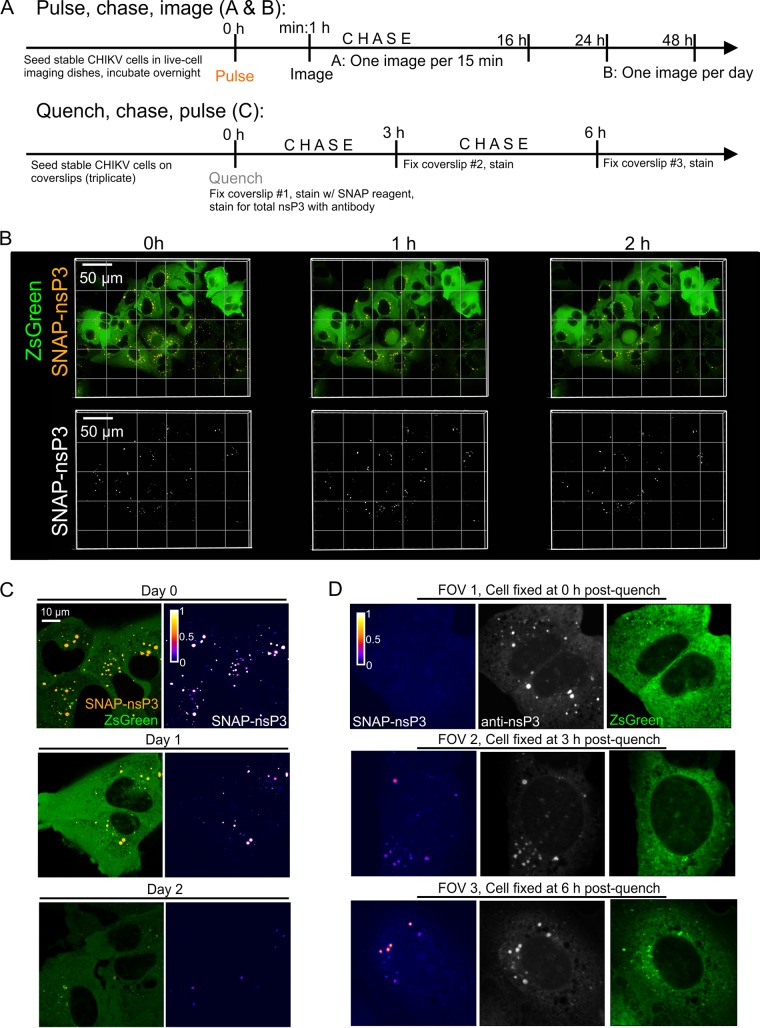
Long-term imaging of SNAP-nsP3 using pulse-chase and quench-pulse-chase approaches. (A) Schematic drawing of pulse-chase image and quench-pulse-chase approaches. “Pulse” refers to labeling with a SNAP-specific dye; “chase” refers to incubation in standard culture medium free of SNAP-specific dyes. (B) Stable CHIKV cells were plated in glass-bottom dishes. Labeling with BG-647-SiR was carried out the following day. Cells were imaged with a Nikon Ti2-E inverted microscope equipped with a light-emitting diode (LED) light source, a 60× oil 1.4-NA objective, and a heated stage insert (set to 37°C with 5% CO_2_). Z-stacks of the same position were taken every 30 min for a total of 16 h. The entire time-lapse recording is also provided in Video S1 in the supplemental material. (C) Cells were prepared as described for panel B but imaged with an Airyscan microscope operated in the Fast Airyscan mode after the final wash (0 h), after 24 h (day 1), and after 48 h (day 2). Cell dishes were returned to a heated incubator after separate FOVs were imaged at each time point. (D) Quench-pulse-chase experiment. Stable CHIKV cells were plated in 24-well plates containing glass coverslips. The next day, nonfluorescent bromothenylpteridine was used to block the reactivity of intracellular SNAP-nsP3. Blocked cells were fixed with 4% formaldehyde at the indicated times postblock (0 h, 3 h, 6 h), and newly synthesized SNAP-nsP3 was stained with BG-647-SiR postfixation. Total nsP3 was stained with an indirect immunofluorescence assay using nsP3-specific antibodies. Stained samples were imaged with an LSM880 system operated in the Fast Airyscan mode. One representative field of view (FOV) is shown from each sample. The same laser power and detector settings were used to image each FOV. Z-stacks were acquired to capture all the granules present within cells. Images are maximum-intensity projections. The SNAP-nsP3 channel was pseudocolored with the Fire lookup table.

SNAP reagents can label live cells, allowing both the analysis of the movement of tagged proteins and pulse-chase studies to examine protein turnover. nsP3-containing granules labeled at the onset of a pulse-chase image experiment (Fig. 5A and B) could be tracked in live-cell imaging experiments over the entire length of a recording that lasted 16 h (Fig. 5B; see Video S1 in the supplemental material). Moreover, stable CHIKV cells still contained “aged” nsP3-containing granules after chase periods of 1 to 2 days (Fig. 5C). The addition of a nonfluorescent SNAP ligand (i.e., quench) in complementary quench-pulse-chase experiments (Fig. 5A) blocked all binding sites of the SNAP-tagged protein pool (Fig. 5D, field of view 1 [FOV1]). After a defined chase period of 3 and 6 h in unlabeled medium, pulsing with the fluorescent SNAP reagent uncovered an unblocked population of nsP3-containing granules, consistent with newly synthesized protein accumulating in granular structures (Fig. 5D, FOV2 and 3).

**FIG 6:**
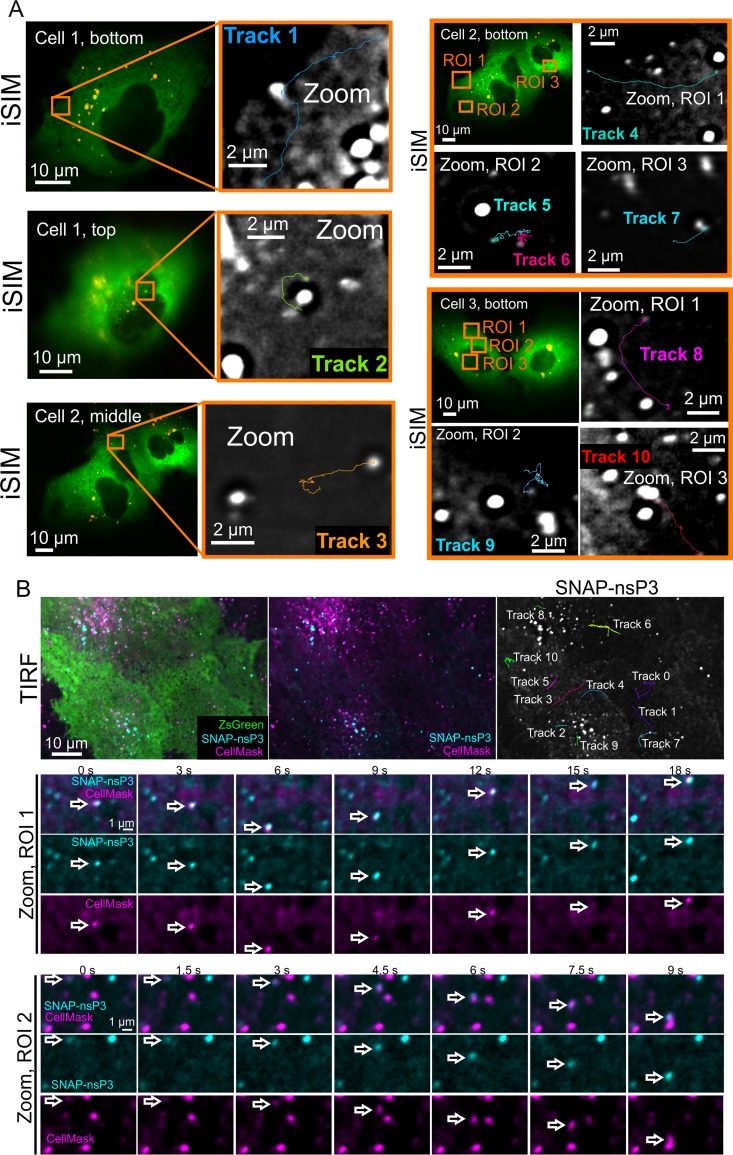
Live imaging of SNAP-nsP3 in stable CHIKV cells showing movement patterns of nsP3-containing granules. (A) Live imaging of SNAP-nsP3 in stable CHIKV cells by instant structured illumination microscopy (iSIM). Entire recordings are also included in Video S2 to S5 in the supplemental material. The two-dimensional (2-D) time-lapse series consisted of 100 to 200 frames. The original 2-D time-lapse series consisted of 100 to 200 frames but were cropped to the relevant frames in zoomed-in views. Images were acquired at intervals of 88 ms. The relative axial position of each ZsGreen-positive cell is indicated as top, bottom, or middle of the cell. The statistics for tracks 1 to 10 are listed in Table 2. (B) Live imaging of SNAP-nsP3 in stable CHIKV cells by total internal reflection fluorescence (TIRF) microscopy. After labeling SNAP-nsP3 with BG-647-SiR (cyan), the plasma membrane was stained with CellMask dye (magenta). Live-cell images were acquired after at least 1 h to allow for internalization of the CellMask dye via membrane-containing vesicles. Entire recordings are also included in Video S6 in the supplemental material. The statistics for tracks 0 to 10 are listed in Table 2. Arrows serve as digital fiducial markers and point toward nsP3- and CellMask-containing structures showing cotrafficking.

To further study the intracellular transport of nsP3-containing granules, we used instant structured illumination microscopy (iSIM) for live-cell recordings at high frame rates (39). iSIM increases spatial resolution by a factor of √2 compared with wide-field microscopy and by a further factor of √2 with postprocessing, while rapid image capture provides the temporal resolution needed for dynamic events within cells. A variety of fast-moving nsP3-containing objects with linear displacements and intermittent bursts of speed were tracked in live-cell recordings (Fig. 6A; Videos S2 to S5). As can be seen in the videos, the movement of these nsP3-containing objects differed from static objects containing nsP3, which were imaged in the same recording. Fast-moving granules containing nsP3 could reach speeds of 0.8 to 5.9 μm/s (Table 2) with net displacements of 9.77 and 10.58 μm measured in the longest tracks (Table 2, tracks 1 and 4). Next, to complement our iSIM live-cell imaging analysis, we took advantage of total internal reflection fluorescence (TIRF) microscopy, which images only fluorescent molecules located close to the glass/specimen interface. TIRF microscopy produced images with good signal-to-noise ratio and reduced the background fluorescence from out-of-focus planes, enabling the observation of nsP3-containing granules located in or close to the plasma membrane (Fig. 6B; Video S6). Tracks analyzed in iSIM and TIRF images revealed similar object displacements and peak velocities (Table 2). Prior to TIRF imaging, we also labeled the plasma membrane with the CellMask orange plasma membrane stain and captured the images at least 1 h after staining to ensure that some of the dye was internalized in membrane-containing vesicles. Multicolor TIRF microscopy then allowed us to visualize the cotrafficking of nsP3- and CellMask-containing structures (Fig. 6B, Zoom, ROI 1 and 2). In summary, the dynamic analysis of nsP3-containing granules showed that they (i) could persist in cells for days, (ii) accumulated newly synthesized protein, (iii) could be classified into static and motile subclasses with characteristic displacements and speeds, and (iv) cotrafficked with membrane-containing structures.

**TABLE 2:** Quantification of tracks

Microscopy type and track no.^*a*^	Start time (s)	End time (s)	Duration (s)	Total displacement (μm)^*b*^	Net displacement (μm)^*c*^	Peak velocity (μm/s)^*d*^
iSIM						
1	0	8	8	14.15	9.77	4.7
2	0	3	3	3.96	1.67	3.1
3	0	13	13	7.82	2.71	1.4
4	0	8	8	12.92	10.58	5.9
5	0	9	9	9.23	2.23	3.8
6	0	9	9	8.80	0.69	3.3
7	0	18	18	3.09	2.1	0.8
8	0	14	14	9.96	5.71	2.8
9	0	13	13	10.45	1.37	2.8
10	0	7	7	5.9	4.65	2.3
TIRF						
0	0	84	84	23.76	3.54	1.2
1	85	120	35	11.58	10.23	1.5
2	83	119	36	4.58	3.62	0.6
3	81	110	30	14.07	12.77	2.3
4	77	88	12	11.05	9.09	1.8
5	23	47	24	11.41	2.33	2.4
6	0	120	120	42.83	6.25	2.5
7	14	26	12	9.76	6.78	1.6
8	103	120	17	4.23	3.55	0.8
9	1	24	23	5.06	3.22	1.1
10	5	120	115	27.12	2.42	0.8

a iSIM, instant structured illumination microscopy; TIRF, total internal reflection fluorescence microscopy.

b Total displacement, the sum of all consecutive displacements in each track, which corresponds to the total distance traveled by the object.

c Net displacement, the distance between the starting and ending positions of each track.

d Peak velocity, highest velocity of the object over the duration of each track.

### Static internal architecture of nsP3-containing granules during persistent replication.

**FIG 7:**
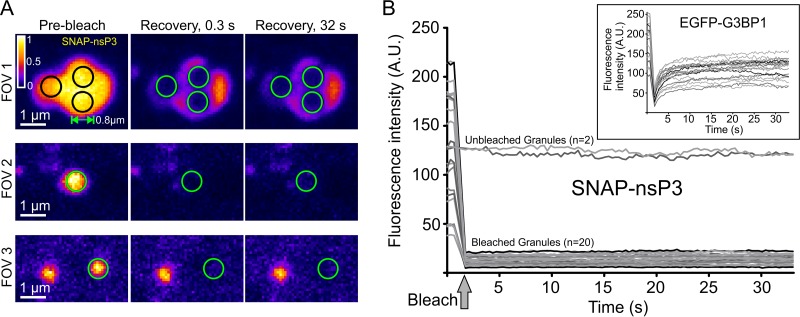
Static internal architecture of nsP3-containing granules. (A) FRAP of stable CHIKV cells stained with BG-TMR-Star. Single-channel images of three FOVs were pseudocolored according to the Fire predefined color map in Icy software. Black and green circles mark photobleached areas (diameters of ∼0.8 μm). Graphs plot the mean fluorescence intensity within each bleached area over time. Intensity values from 20 ROIs were pooled from a total of 10 FOVs. Two unbleached ROIs of the same size were included as controls. Insets show FRAP experiments of uninfected HuH-7 cells overexpressing an EGFP-G3BP1 plasmid. Ten FOVs of cells overexpressing EGFP-G3BP1 granules were selected, and 20 ROIs were pooled to create graphs. All FRAP experiments were done with an LSM700 microscope with a Plan-Apochromat 63× 1.4-NA oil objective. Images of cells were recorded every 0.32 s after the photobleaching for a total of 100 cycles.

To determine the dynamic behavior of nsP3 in granules, we performed fluorescence recovery after photobleaching (FRAP) experiments. Stable CHIKV cells were labeled with BG–6-carboxytetramethylrhodamine (TMR)–Star, defined regions with diameters of about 0.8 μm were photobleached, and fluorescent recovery was measured within each region of interest (Fig. 7A). No fluorescence recovery occurred over the duration of the experiment (Fig. 7A and B), suggesting that nsP3 remained fixed within the granular architecture and did not undergo dynamic exchange within each granule or with the surrounding cytoplasm. In contrast, SGs formed during overexpression of an enhanced green fluorescent protein (EGFP)-G3BP1 fusion in uninfected HuH-7 cells showed a characteristic rapid recovery of fluorescence after the photobleaching (Fig. 7B, inset), consistent with G3BP1 rapidly shuttling into and out of SGs.

**FIG 8:**
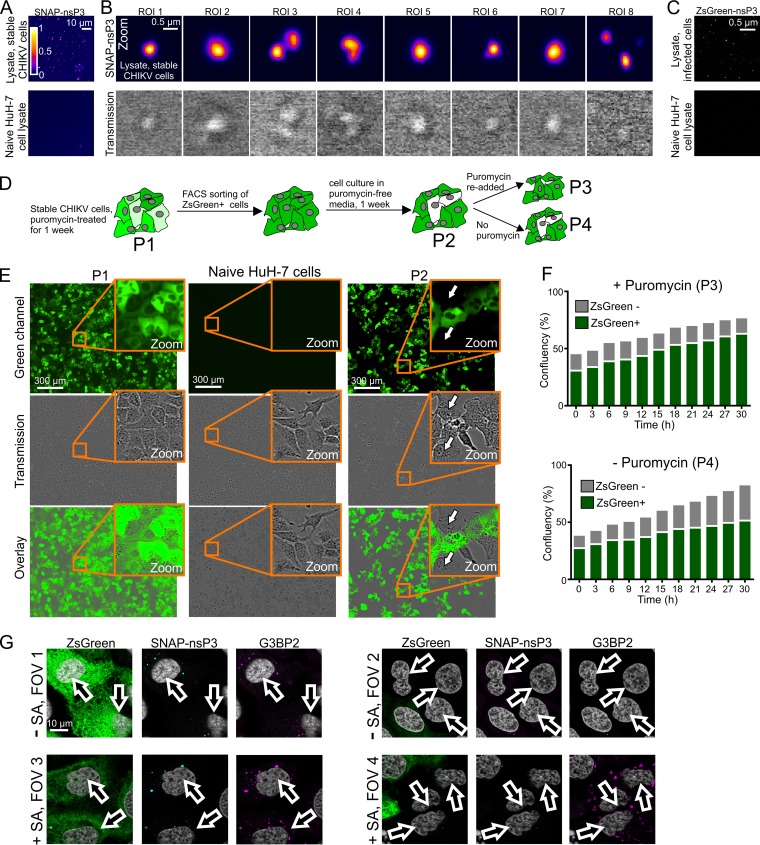
(A) Cell lysates from stable CHIKV cells. Live cells were stained with BG-TMR-Star and lysed with Glasgow lysis buffer. The lysate was then bound to plastic chamber slides overnight and imaged the following day. Images were acquired with an LSM880 microscope operated in Fast Airyscan mode. Cell lysates from uninfected, naive HuH-7 cells are shown as a control. (B) Zoomed-in views of the sample shown in panel A, providing higher-magnification views of nsP3-containing granules and corresponding bright-field images (transmission). (C) Cell lysates from HuH-7 cells infected with CHIKV^ZsGreen-P3^. Samples were prepared as described for panel A and imaged in the green channel. (D) Schematic overview of different populations (P1 to P4) obtained during culture of stable CHIKV cells. (E) Wide-field microscopy of stable CHIKV cells passaged for 1 week in the absence of puromycin (P2). Naive HuH-7 cells and cells treated for 1 week with puromycin (P1) are shown as controls. Images were obtained with an IncuCyte Zoom live-cell imaging system. (F) Effect of puromycin treatment on mixed populations containing both ZsGreen-positive and ZsGreen-negative cells. Confluence of the two populations was determined from images taken with an IncuCyte Zoom live-cell imaging system. (G) Effect of sodium arsenite treatment on mixed populations containing both ZsGreen-positive and ZsGreen-negative cells. To induce cellular stress granules, sodium arsenite was added for at least 30 min. Cells were fixed and then stained for SNAP-nsP3 (cyan) and G3BP2 (magenta). Stained cells were imaged by Airyscan microscopy. FOVs 1 and 3 were centered on cells expressing ZsGreen, whereas FOVs 2 and 4 focused on cells that were ZsGreen negative.

Previously, G3BP1-containing SGs were shown to be stable in cell lysates, suggesting that stable core structures make up these membrane-less organelles (30). To further test the stability of nsP3-containing granules, we microscopically examined lysates of stable CHIKV cells (Fig. 8A). Bright-field images of cell lysates indicated the presence of refractive granules, while fluorescence microscopy identified granules that had incorporated the BG-TMR-Star label (Fig. 8B). We also confirmed that the stability of nsP3-containing granules was not unique to stable CHIKV cells, as we could also detect ZsGreen-positive granules following lysis of cells infected with CHIKV^ZsGreen-P3^ (Fig. 8C).

In stable CHIKV cells, ZsGreen serves as a fluorescent marker of subgenomic replicon-RNA synthesis (Fig. 1A, cartoon). The green fluorescence also allowed us to use fluorescence-activated cell sorting (FACS) to eliminate any ZsGreen-negative cells. Although stable CHIKV cells maintained high levels of SNAP-nsP3 and ZsGreen for up to 2 months, cells with reduced or undetectable ZsGreen fluorescence accumulated in the absence of puromycin selection after only 1 week of culturing (Fig. 8D and E). These cells were sensitive to puromycin (Fig. 8D and F), suggesting that they no longer harbored the replicon. To test whether ZsGreen-negative cells that had emerged during culturing in puromycin-free medium regained some of the characteristics of uninfected cells, we subjected a mixed population (with both ZsGreen-positive and -negative cells) to a restress experiment with sodium arsenite. Consistent with previous experiments that examined the effects of “restressing” alphavirus-infected cells (17, 40), ZsGreen-positive cells sequestered G3BP2 into nsP3-containing granules even in the absence of sodium arsenite (Fig. 8G, FOV1), and new G3BP2-containing granules were absent after arsenite-induced stress (Fig. 8G, FOV3). In contrast, cells lacking ZsGreen did not have any G3BP2-containing granules in the absence of arsenite stress (Fig. 8G, FOV2) but were able to form G3BP2-positive clusters after arsenite treatment (Fig. 8G, FOV4). Therefore, the renewed ability to respond to arsenite-induced stress was associated with a loss of viral replication and nsP3-containing granules.

## DISCUSSION

The objectives of this study were, first, to characterize the interaction between CHIKV nsP3 and cellular components during persistent replication and, second, to evaluate the persistence of cytoplasmic granules composed of viral and cellular proteins. To achieve these objectives, we expanded the utility of a noncytotoxic replicon by combining it with SNAP tag-based fluorescent labeling and subdiffraction multicolor microscopy to provide unprecedented insights into the substructure of persistent nsP3-G3BP-containing granules. These studies revealed their relationship with dsRNA, nsP1-positive structures, and the nuclear membrane. Examining the dynamics of nsP3-containing granules uncovered a stable population of nsP3-containing granules along with a subclass of nsP3-positive structures trafficking through the cell cytoplasm. Importantly, we observed that nsP3-containing granules lacked a dynamic internal architecture and remained stable in cell lysates. Lastly, we showed that the ability to respond to oxidative stress was associated with the loss of CHIKV replication and nsP3-containing granules.

### Stable CHIKV cells as a versatile tool for studying cytoplasmic nsP3-containing granules.

Previous reports on noncytotoxic Old World alphaviruses elucidated the relationship between cytotoxicity, nsP2, and viral genome replication (28, 41–43). The typical cytotoxicity of CHIKV replicons precluded long-term studies of a previously described SNAP-tagged replicon. We have now overcome this limitation with a new HuH-7 cell line that harbors replicating CHIKV replicon RNA and encodes both SNAP-tagged nsP3 and ZsGreen as a genetic reporter for subgenomic replicon RNA. Whether this replicon establishes persistent replication only in specific cell types, as has been observed for other noncytotoxic replicons (28, 41), remains to be determined. We found that the SNAP-tagged replicon also persisted in C2C12 mouse myoblasts, albeit less efficiently.

To our knowledge, the system presented here is the first to allow intracellular tracking of nsP3 during persistent replication of CHIKV RNA in a replicon system. A similar accumulation of nsP3 in cytoplasmic granules occurs in transient replicons (16, 26, 27) and during late stages of infection (24, 25, 27). Strikingly, SNAP-nsP3 in stable CHIKV cells did not form rod-like structures, which were observed in cells infected with CHIKV^SNAP-P3^. Rod-like structures appear not only during transient replication in HuH-7 cells (26) but also in other cell types (mouse myoblasts, glial cells, dermal fibroblasts) during transient replication and late stages of infection (25, 27). Moreover, mutagenesis of nsP3's C-terminal domain results in the formation of long rod-like structures (16, 44). In contrast, CHIKV nsP3 preferentially forms granules in specific cell types, such as human muscle and epithelial cell lines (27). Surprisingly, infection with CHIKV^ZsGreen-P3^ was not associated with the presence of rod-like structures. However, we cannot rule out the possibility that rod-like structures only form transiently and are no longer present at the observed time point.

Nonetheless, the lack of rods was not accompanied by a reduction in infectious titers. Thus, our results suggest that the ability to form rod-like structures can be affected by the sequence of the inserted tag in the C-terminal domain (SNAP versus ZsGreen) but also by whether nsP3 is expressed during persistent replication or infection. Interestingly, the noncytotoxic replicon also encodes a leucine residue instead of isoleucine at position 175, in a presumed unstructured region between predicted domains of nsP3 (28). Although this mutation may primarily stabilize replication complexes in conjunction with other noncytotoxic mutations (28), we do not know yet whether it affects the formation of rod-like structures. Taken together, SNAP-nsP3 can form a cytoplasmic mixture of rod-like and granular structures during CHIKV infection, but only granules persist in cells that persistently replicate CHIKV replicon RNA.

### Persistence of nsP3-G3BP-containing granules within a microenvironment containing dsRNA, nsP1, and cellular markers.

Subdiffraction multicolor microscopy of stable cells revealed that nsP3-containing granules were (i) G3BP1 and G3BP2 positive, (ii) juxtaposed to dsRNA foci and nsP1-positive structures, (iii) associated with the nuclear membrane, and (iv) proximal to Nup98-positive organelles. Alphavirus nsP3 forms cytoplasmic granules with vertebrate G3BP1/2 and the mosquito homolog Rasputin (16, 17, 21, 24, 25, 45–47). The noncytotoxic replicon preserved this interaction in cytoplasmic granules whose diameters and protein contents varied. Moreover, Airyscan microscopy allowed us to address the internal substructure of larger granules (>1 μm), which had detectable differences in fluorescence intensity within the granule, suggesting intragranular variations in the density of nsP3. In the future, stochastic optical reconstruction microscopy (STORM), which can provide an even higher resolution to Airyscan microscopy, may be necessary to reveal the detailed substructure of smaller granules (<500 nm). For example, STORM revealed that G3BP-containing SGs had stable core structures with diameters of ∼200 nm (30).

Multicolor Airyscan microscopy provided a convenient workflow to examine ZsGreen-expressing stable cells for interactions between nsP3, dsRNA, and nsP1. Alphavirus nsP1 can bind membranes (48, 49) and may use its membrane-binding domain to tether replication complexes to cellular membranes (50). During infection of the related Semliki Forest virus (SFV), nsP1 colocalizes with G3BPs in putative replication complexes (17). However, the nsP1-nsP3 and nsP1-G3BP associations could not be clearly detected during transient CHIKV replication and CHIKV infection (16, 25, 26). We were able to image a partial overlap of nsP1-positive structures with nsP3 granules in stable cells. Occasionally, nsP1 coated ring-like structures, which may represent virus-induced membranous organelles. Furthermore, we could detect dsRNA-positive foci in contact with nsP3-containing granules. Large cytoplasmic G3BP-nsP3 structures contain viral genomic RNA but not dsRNA, and these complexes grow over the course of an infection (24). During SFV infection, replication complexes initially form at the plasma membrane within so-called spherules, which have a characteristic bulb shape and a diameter of about 50 nm. Later, spherules are internalized and incorporated in large intracellular cytopathic vacuoles, which are derived from endolysosomal membranes and about 0.6 to 2 μm in diameter (51–54). Replication complexes are comprised not only of the complementary negative strand but also the full-length positive strand and the subgenomic mRNA. However, negative-strand synthesis occurs only during the first few hours of SFV infection (55, 56). During Sindbis virus (SINV) infection, dsRNA intermediates are packed into membrane spherules at the plasma membrane and also contain nsP1 early during infection (2 h), whereas nsP1-nsP3-dsRNA cytoplasmic complexes appear later in infection (36). Thus, nsP3 structures that are associated with dsRNA and ring-like structures of nsP1 in this study may be related to cytopathic vacuoles. The fraction of nsP3 associated with active RNA replication complexes varies between alphaviruses (reviewed in reference 57), and a smaller fraction of nsP3-containing structures colocalizes with dsRNA in CHIKV-infected cells than in SFV-infected cells, where most of the staining colocalizes. Moreover, replication complexes are efficiently internalized from the cell periphery for SFV but not CHIKV, and this reduction in CHIKV replication complex internalization correlates with a reduced stimulation of the prosurvival PI3K-Akt-mTOR pathway in comparison to that in SFV infection. Hence, persistent replication of the noncytotoxic replicon may also be associated with an increased internalization of membrane-bound replication complexes and increased colocalization of large, cytoplasmic nsP3-containing granules with dsRNA. Ultimately, correlative light and electron microscopy (CLEM) of stable CHIKV cells can elucidate the ultrastructure of nsP3-containing granules and their relationship with membranous organelles, as was done for SFV (58). Stable CHIKV cells offer particular advantages during CLEM sample preparation: (i) tetramethylrhodamine-coupled SNAP ligands are compatible with CLEM approaches (59), (ii) every puromycin-selected cell is guaranteed to harbor the replicon, and (iii) ZsGreen fluorescence marks the cytoplasm of imaged cells.

We also captured high-resolution images of an association between nsP3-containing granules and the nuclear membrane. Moreover, we investigated the previously unexplored relationship between nsP3 and the nucleoporin Nup98. Little is known about the nuclear transport of nsP3, while the localization of nsP2 to the nucleus is well documented (16, 46, 60, 61). Intriguingly, a role for G3BP1 as a nuclear transport factor has been proposed, and SINV nsP3 has been identified at the nuclear membrane (21). Our results imply that nsP3-containing granules are associated with a nucleoporin during persistent replication and may connect to RNA transport pathways at the nuclear membrane. Viral proteins that bind to Nups or RNA transport factors have been shown to stimulate remodeling of the nuclear membrane and affect the nuclear transport of cellular mRNA and proteins (62, 63). During SFV infection, many nuclear proteins relocate to the cytoplasm, where they play both proviral and antiviral roles (64). We also observed an association of nsP3 granules with cytoplasmic Nup98. During hepatitis C virus (HCV) infection, cytoplasmic nucleoporins accumulate at sites rich in viral proteins, including virus-induced membranous organelles and cytosolic lipid droplets (65, 66). In summary, Nups may play a role in persistent replication of CHIKV, which could hijack the physiological functions of nucleoporins to transport CHIKV nonstructural protein components, mRNA, viral RNA, or cellular proteins. Our data warrant a further investigation of this hypothesis.

### Stable CHIKV cells contain a mixture of static and dynamic nsP3-containing granules, which lack a dynamic internal architecture and are stable in cell lysates.

Self-labeling enzyme tags such as the SNAP tag provide experimental control over the time of labeling, thereby allowing us to study protein turnover. nsP3-containing granules were stable for hours and persisted for days. Granules were also the site where newly synthesized nsP3 accumulated. Thus, old and new populations of nsP3 may continuously mix within cytoplasmic granules, as was seen during transient replication (26). Live-cell microscopy also provided the first real-time tracking of CHIKV nsP3-containing granules and in-depth view of granule dynamics in mammalian cells. Previous live-cell microscopy revealed three subclasses of nsP3 structures during SFV infection: (i) small, nonacidic, nsP3-positive vesicles undergoing multidirectional and short-distance (2-μm) movement reminiscent of actin-based movement, (ii) large, acidic vesicles displaying less-frequent jumps over distances of >10 μm, and (iii) large, acidic vesicles that were immobile and concentrated in the perinuclear area (51). Blebbistatin, an inhibitor of the actin motor protein myosin II, inhibited the dynamic movements of small vesicles, while nocodazole, a tubulin-disrupting agent, inhibited saltatory movements (51). We report similar movement patterns, including (i) the presence of immobile granules within perinuclear regions and (ii) granules moving over short (1- to 3-μm) and long (>4-μm) distances at maximum speeds between 0.8 and 5.9 μm/s. We also visualized cotrafficking of nsP3- and membrane-containing structures, which suggests that nsP3 moves through the cell by hijacking components of the cellular secretory machinery. In the future, stable CHIKV cells can provide invaluable real-time insight into interactions between CHIKV and the host through multicolor imaging of ZsGreen, far-red-fluorescent SNAP-nsP3 labeling, and a third, blue or red, fluorescent marker.

FRAP experiments revealed the static internal architecture of nsP3-containing granules, whereas arsenite-induced G3BP-containing granules had a fluorescence recovery similar to that seen in human osteosarcoma cells (67). The absence of a rapid exchange in CHIKV-induced granules implies that nsP3 may play a role that differs biochemically from the dynamic role of G3BP1 in SGs (30, 31, 68). For example, nsP3 may create a scaffold similar to the one formed by Fas-activated serine/threonine kinase (FASTK) in SGs (68). Although we cannot rule out that G3BP1 or G3BP2 shuttles in and out of nsP3-containing granules, we predict that G3BP1/2 would be similarly fixed in granules: nsP3 completely overlapped G3BP, and nsP3-containing granules were stable enough to be preserved in cell lysates. Moreover, previous studies demonstrated that alphavirus nsP3-G3BP-containing granules lack canonical SG markers (16, 17) and remain stable during cycloheximide treatment (16), which dissolves SGs (69). FRAP experiments of membrane-associated foci containing nonstructural proteins of another RNA virus, HCV, also found a limited exchange between clusters of nonstructural proteins and the periphery (70–72). Thus, some of the nsP3 structures may represent cytopathic vacuoles, in which nsP3 has a limited exchange with the surrounding cytoplasm.

Unlike cytopathic vacuoles, which would be sensitive to detergents, a population of nsP3-containing granules was detergent resistant and stable in cell lysates. This persistence in lysates mimics that of mammalian SG cores, where a dynamic shell around core structures gives SGs biochemical qualities akin to liquid-liquid phase separations (30). We propose that similar stable core structures might make up nsP3-G3BP-containing granules. Recent studies show that environmental conditions can cause proteins bearing intrinsically disordered protein regions to undergo liquid-liquid phase separation and assemble droplets, hydrogels, and aggregates; this concentration of proteins into discrete subcellular domains appears to be essential for cellular metabolism and stress responses (for a recent review, see reference 73). In turn, defects in the regulation of such membrane-less organelles could impair cellular functions, alter stress responses, and form the basis of pathogenic inclusions linked with neurodegenerative disease (74). Intriguingly, the C terminus of alphavirus nsP3 itself is unstructured, which is a prerequisite not only for proteins to undergo phase separation but also to form more solid gel-like granules. Moreover, viral genomic RNA colocalizes with nsP3-G3BP (24), providing evidence that nsP3-G3BP-containing granules are made up not only of protein but also of RNA, a key component of cellular ribonucleoprotein granules (for a review on ribonucleoprotein granules, see reference 75). The link between liquid-liquid phase separation, membrane-less organelles, stress responses, and toxic protein clusters forms the basis of a new hypothesis that nsP3-containing granules can perturb cellular responses to environmental conditions. However, more experiments are needed to (i) further characterize persistent nsP3-containing granules biochemically, (ii) identify other cellular or viral proteins within granules, and (iii) induce granular disassembly. Clearing cells of these stable cytoplasmic complexes could be essential for preventing any toxicity that emerges during prolonged exposure to CHIKV proteins. Moreover, directly targeting persistent nsP3-containing granules could lead to new approaches to combat chikungunya virus infections. Cells that had turned ZsGreen negative during culturing in puromycin-free medium were not irreversibly perturbed but rather had regained the ability to form SGs in response to arsenite treatment.

In summary, our results present the first evidence that granules containing the viral protein nsP3 and cellular protein G3BP persist in human cells with autonomously replicating CHIKV replicon RNA. Generation of a cell line harboring a persistently replicating SNAP-tagged replicon and advances in microscopy technology allowed us to reveal interactions between SNAP-nsP3, viral components (nsP1, dsRNA), and the nuclear membrane. Overall, nsP3-containing granules were stable, differed in their mobility, lacked a dynamic internal architecture, and were stable in cell lysates. These findings may also have clinical relevance, as CHIKV can cause chronic infection and persist in various cell types, such as macrophages, muscle, and liver cells. However, whether prolonged exposure to nsP3-containing granules causes pathogenic changes within the cell and can contribute to chronic chikungunya disease remains to be determined. Lastly, the reagent presented in this study adds a new dimension for future explorations of host-pathogen interactions, in particular as they relate to nsP3, and for the search for inhibitors that specifically target nsP3.

## MATERIALS AND METHODS

### CHIKV constructs.

The replicon CHIKVRepRLuc-FL-5A-PG-IL was described previously and allows for stable, noncytotoxic growth in HuH-7 cells (28). It contains a cassette encoding a puromycin-*N*-acetyltransferase (Pac)-FMDV 2A autoprotease-ZsGreen fusion under the control of the subgenomic promoter. In the CHIKVRepRLuc-FL-5A-PG-IL replicon, a Renilla luciferase (Rluc) flanked by SpeI restriction sites was inserted into nsP3. The SNAP-tagged replicon, which has a SNAP sequence (also flanked by SpeI restriction sites) inserted into nsP3, has also been described previously (26). The parental replicon used in the generation of the SNAP-tagged replicon was originally assembled from DNA constructs containing the CHIKV replicon cDNA from the LR2006 OPY1 strain, which was isolated from the serum of a febrile patient traveling from La Réunion (76); cDNA fragments (Geneart) were synthesized based on the published sequence of the LR2006 OPY1 strain and assembled *in vitro* to generate fully synthetic replicons. To generate a noncytotoxic SNAP-tagged replicon (CHIKVRepSnap), we ligated a DNA fragment corresponding to the region encoding the SNAP tag (excised by SpeI digestion of SNAP-tagged nsP3) to SpeI-digested CHIKVRepRLuc-FL-5A-PG-IL vector.

Restriction site cloning via SpeI was used to replace a gene encoding the green fluorescent ZsGreen protein (originally derived from an Anthozoa species of reef corals [77]) with the SNAP sequence, in the context of an infectious CHIKV virus (CHIKV^ZsGreen-P3^). This infectious clone was synthesized previously based on the sequence from CHIKV LR2006 OPY1 (43).

CHIKV constructs were verified by DNA sequencing of nsP3 regions (to confirm correct orientation of SNAP tag after ligation at SpeI sites) and the subgenomic region, as well as analysis of EcoRI/BamHI restriction digest patterns to test for the overall integrity of CHIKV replicons and infectious constructs.

### Cells, media, transfection, and infection.

HuH-7 cells were maintained in complete medium (Dulbecco's modified Eagle's medium supplemented with fetal calf serum, penicillin, streptomycin, nonessential amino acids, and HEPES buffer) as described previously (26). HuH-7 is a well-differentiated hepatocyte-derived cellular carcinoma cell line taken from the liver tumor of a male Japanese patient in 1982 (78); these cells were from John McLauchlan (Centre for Virus Research, Glasgow). Growth medium supplemented with puromycin (final concentration, 5 μg/ml) was used for antibiotic selection.

### *In vitro* transcription and electroporation of CHIKV RNA.

Plasmids containing cDNA of SNAP-tagged noncytotoxic CHIKV replicon were linearized by NotI digestion. Purified DNA was used as the template for an *in vitro* transcription reaction using the mMESSAGE mMACHINE SP6 transcription kit (Ambion). RNA was purified with the PureLink RNA minikit (Thermo Fisher Scientific) and stored in aliquots of distilled water at −80°C until the day of electroporation. RNA was transfected into cells via electroporation as described before (26). Electroporated cells were seeded in 10-cm dishes. Cells were incubated in puromycin-free medium for a minimum of 2 days before starting puromycin selection. During puromycin selection, cells were monitored with a wide-field fluorescence microscope and a fluorescein isothiocyanate (FITC) filter setup for ZsGreen fluorescence. After ZsGreen-positive cells reached a high proportion (2 to 5 days), cells were expanded in puromycin-free medium. Heterogeneous populations of ZsGreen-positive cells, which we call stable CHIKV cells, were collected from confluent T75 flasks to make frozen cell stocks in fetal calf serum supplemented with 10% dimethyl sulfoxide (DMSO) (about 2 weeks after electroporation). At the same time, stable CHIKV cells were passaged under standard cell culture conditions and used in microscopy experiments. To study the appearance of a subpopulation of ZsGreen-negative cells, a pure population of ZsGreen-positive cells was obtained with fluorescence-activated cell sorting (FACS) of live cells. Cell populations were sorted with a FACsMelody instrument (BD Biosciences) based on green fluorescence (488-nm laser and 527/32 filter). Only singlets were picked to avoid artificially high fluorescence. Following a cell sort of 1 million cells, cells were plated into a T25 flask and allowed to expand in puromycin-free medium to allow for the appearance of ZsGreen-negative cells.

For infection experiments, plasmids containing cDNA of CHIKV^SNAP-P3^ and CHIKV^ZsGreen-P3^ were linearized by NotI digestion. *In vitro* transcription was carried out as described above. HuH-7 cells were harvested from T175 flasks, electroporated at 0.5 × 10^7^ cells/ml using a square-wave protocol at 260 V for 25 ms, seeded into T175 flasks, and allowed to incubate for multiple days. Supernatants were frozen and used as virus stocks. The working stock of CHIKV was plaque titrated in BHK-21 cells (ATCC CCL10). For microscopy analysis of viral infection, this working stock was added to naive HuH-7 cells at a multiplicity of infection (MOI) of 10 and fixed 24 h later. To compare the multiplication of ZsGreen- and SNAP-tagged virus, HuH-7 cells were infected with viral stocks at the same MOI; supernatants were collected at 24, 48, and 72 h postinfection and plaque titrated in BHK-21 cells.

### Primary and secondary antibodies.

Polyclonal anti-G3BP2 was obtained from Bethyl Laboratories, mouse anti-G3BP1 antibody was from BD Biosciences, and antibodies detecting Nup98 were from Cell Signaling. For immunofluorescence labeling of dsRNA, mouse monoclonal anti-dsRNA (J2; Scicons) was used. J2 specifically recognizes dsRNA of more than 40 bp in length (79). Polyclonal rabbit antibodies against CHIKV nsP3 and nsP1 were produced in-house (Merits laboratory). Whole species-specific IgG secondary antibodies were either anti-rabbit Alexa Fluor 594-conjugated IgG (Thermo Fisher Scientific) (Fig. 2 and 4), anti-rabbit DyLight 405 IgG (to detect nsP1 in Fig. 3), or anti-mouse Alexa Fluor 594-conjugated IgG (to detect J2 in Fig. 3).

### Intracellular SNAP tag staining.

To stain intracellular SNAP-tagged proteins with the standard protocol, benzylguanine (BG), conjugated to fluorophores (silicon rhodamine [SiR], or TMR-Star, commercially available as SNAP-Cell 647-SiR and SNAP-Cell TMR-Star [NEB]), was added to live cells and incubated for at least 15 min at 37°C, 5% CO2. This was followed by three washes in complete medium and an extended incubation in complete medium for at least 30 min to remove background fluorescence. For fixed-cell microscopy analysis shown in Fig. 1, cells were fixed at room temperature with 4% formaldehyde for 30 min. Cells were then counterstained with 4′,6-diamidino-2-phenylindole (DAPI) and mounted onto glass slides by the addition of ProLong diamond antifade mountant (Thermo Fisher Scientific).

### IFAs.

For indirect immunofluorescence assay (IFA) and staining with G3BP1, G3BP2, or J2 antibodies, formaldehyde-fixed cells were permeabilized with 100% methanol for 10 min at −20°C. For all other antibodies, cells were permeabilized with a buffer containing 5% fetal calf serum and 0.3% Triton X-100. Cells were incubated with primary antibody solution containing 1% bovine serum albumin (BSA) overnight at 4°C, except the mouse J2 antibody, which was incubated for 2 h at room temperature in diethylpyrocarbonate-treated phosphate-buffered saline (PBS). After three washes in PBS, secondary antibody (anti-rabbit Alexa Fluor 594-conjugated IgG or anti-mouse Alexa Fluor 594-conjugated IgG; Molecular Probes) was added. For nsP3/J2/nsP1 triple staining, rabbit nsP1 antibody was added overnight at 4°C to cells already stained with BG-647-SiR (benzylguanine-silicon-rhodamine) and mouse J2. The following day, cells were washed three times in PBS, and secondary antibody (anti-rabbit Alexa Fluor DyLight 405) was added. These cells were not counterstained with DAPI. However, where indicated (Fig. 1, 2, and 4), DAPI was added to visualize nuclei. Coverslips were mounted onto glass slides by the addition of ProLong diamond antifade mountant (Molecular Probes).

### Pulse-chase and quench-pulse-chase experiments.

For long-term pulse-chase experiments (Fig. 5B and C), stable CHIKV cells were plated in 35-mm glass-bottom dishes with a no 1.5 gridded coverslip (Nunc); labeling with BG-SiR was carried out the following day using the live-cell protocol described above. Live-cell imaging solution (supplemented with HEPES, 10% FBS, nonessential amino acids, and ProLong live antifade reagent) was added after the final wash. A Nikon Ti2-E inverted microscope was used to image the turnover of SNAP-nsP3 over 16 h. To image live cells after 24-h and 48-h chase periods, an LSM880 imaging system was operated in Fast Airyscan mode. Different fields of view were taken with the same imaging settings as those for the 0-h time point.

For quench-pulse-chase experiments, stable CHIKV cells were plated in 24-well plates containing 13-mm glass coverslips. The next day, 10 μM nonfluorescent bromothenylpteridine (SNAP-Cell block; NEB) was used to block the reactivity of intracellular SNAP-nsP3 in stable CHIKV cells. After a 45-min incubation, blocked cells were washed three times with complete medium, followed by a 30-min incubation in complete medium. Cells were fixed with 4% formaldehyde at the indicated times (Fig. 5) postblock (0 h, 3 h, 6 h), and newly synthesized SNAP-nsP3 was stained with BG-SiR. Total nsP3 was stained with a rabbit antiserum against nsP3 and dye-conjugated secondary antibodies (anti-rabbit Alexa Fluor DyLight 405). Stained coverslips were mounted onto glass slides in ProLong diamond (Molecular Probes), and Z-stacks were acquired with an LSM880 system (Zeiss) operated in the Fast Airyscan mode. A Plan-Apochromat 63×/1.4 oil Ph3 M27 objective was used for these experiments.

### Subdiffraction light microscopy.

An LSM880 upright confocal microscope with Airyscan (Zeiss) was used to acquire subdiffraction microscopy images as described previously (26, 80). This microscope provides a maximum lateral resolution of 140 nm and an axial resolution of 400 nm for a fluorophore emitting at 480 nm. Z-stacks were acquired with a 63×/1.4NA Plan-Apochromat oil objective at a step size of 0.16 μm. Pixel size was 40 nm by 40 nm by 160 nm. Sequential scans (scan zoom = 4 in frame mode, 1-s frame time, averaging set to 1 or 2) were acquired in four channels, as follows: channel 1 = 633 nm laser, channel 2 = 561 nm laser, channel 3 = 488 nm, channel 4 = 405 laser. Z-stacks in Fig. 2, 5C, 5D, 8A, 8B, and 8G and single-slice images of live cells (Fig. 2F) were acquired with the Fast Airyscan mode. To increase signal-to-noise ratio and resolution, image stacks were processed by Airyscan processing within Zen Black. Single-slice images were extracted to produce panels in Fig. 2, 3, and 4.

### Live-cell microscopy of stable CHIKV cells.

Live-cell wide-field imaging in Fig. 5B was done with a Nikon Ti2-E inverted microscope equipped with a Lumencor Spectra X LED light source, CFI Plan Apo Lambda 60× oil/1.4NA objective, a photometric Prime 95B sCMOS monochrome camera, and a heated stage insert (set to 37°C with 5% CO_2_). Z-stacks were taken every 30 min for a total of 16 h. Cells were grown in 35-mm glass (no. 1.5)-bottom dishes with a 27-mm viewing area (Nunc). Stable CHIKV cells were stained with BG-647-SiR and then maintained at 37°C in an optically clear, physiological, and CO_2_-independent imaging buffer (Molecular Probes; live-cell imaging solution supplemented with 10% fetal calf serum, nonessential amino acids, and buffered with 10 mM HEPES). To suppress photobleaching, ProLong live antifade reagent was added according to the manufacturer's instructions (Molecular Probes).

A home-built instant structured illumination microscope (iSIM) was used to acquire additional subdiffraction time-lapse series at high frame rates (one image every 88 ms) (Fig. 6A and B). This instrument is fitted with an Olympus water immersion objective 1.2-numerical aperture (NA) UPLSAPO 60XW and 488-nm and 561-nm lasers (81). Stable CHIKV cells were stained with red-fluorescent BG-TMR-Star before image acquisition. The heated stage was set to 37°C. The same live-cell imaging medium described above was used, supplemented with ProLong live antifade reagent. Regions of interest were found using the live iSIM display in the green channel (ZsGreen) to avoid bleaching of the red channel (nsP3). A single-slice two-color image of the green and red channels was taken as a reference. Two-color reference images were processed to remove striped scanning artifacts (39) with the stripes filter in ImageJ plugin Xlib (82). The Z-position corresponded to either the bottom, middle, or top of the cell. Time-lapse series were acquired by taking images of the red channel at intervals of 88 ms for 100 to 200 cycles. Cropped ROIs from these time-lapse series were processed using the Richardson-Lucy algorithm in the ImageJ plugin DeconvolutionLab (six iterations) (83) and a Gaussian filter (σ = 1 pixel). Image contrast was adjusted for each cropped time-lapse series within the Icy (http://icy.bioimageanalysis.org) platform (84) by dragging the adjustable bounds of the histogram viewer, which enhances the contrast in the selected channel without altering the data (84). A viewing range that provided the best contrast for the moving objects within the time lapse was selected.

For TIRF (total internal reflection fluorescence) microscopy, a Ti2-E inverted microscope equipped with an LU-N4 laser bed (405, 488, 561, 647), a CFI Apochromat SR TIRF 100× oil 1.5-NA objective, and a photometric Prime 95B sCMOS monochrome camera was used. Stable CHIKV cells were stained with BG-SiR. The plasma membrane was stained with red-fluorescent CellMask orange (Molecular Probes). Three-color images (green, red, far-red) of the ZsGreen, CellMask orange, and BG-SiR signals were taken. Time-lapse images were acquired at least 1 h after staining with CellMask orange to allow for internalization of this plasma membrane-specific dye. The total duration of the three-color TIRF time lapse was 2 min, with 167 cycles.

Images of ZsGreen-positive and ZsGreen-negative cells (Fig. 8E) were acquired with an IncuCyte Zoom system (Essen BioScience), which consists of an automated phase-contrast and fluorescence microscope housed within a humidifying incubator connected to a 5% CO_2_ line. For quantification of ZsGreen-positive and ZsGreen-negative populations in response to puromycin (Fig. 8F), cells cultured for at least 1 week in puromycin-free medium were plated in six-well plates. The next day, medium was changed to either puromycin-containing or puromycin-free medium and microscopy images were taken at 3-h intervals for a total time of 30 h. In each well (well 1, puromycin treated; well 2, puromycin free), the system was set to take nine images with the following software settings: Nikon 10× objective, dual-color-filter module (model 4459), two image channels (green and phase with 1,392 by 1,040 pixels at 1.22 μm per pixel, acquisition times of 400 ms and 1,000 ms, respectively). Cell confluence (in %) and green object confluence (in %) were determined with the basic analyzer module of the IncuCyte Zoom software.

### FRAP analysis.

Stable CHIKV cells, stained with BG-TMR-Star with the live-cell protocol, were used for experiments imaging SNAP-nsP3. An LSM700 imaging system (Zeiss) was used for FRAP experiments. Circular bleach areas were drawn within the Zen Black software (diameter of about 0.8 μm). Analyzed ROIs were pooled from recordings of 10 FOVs. One reference region of identical size was drawn over a granule and left unbleached. Another reference region was drawn within the cytoplasm to measure fluorescence background. Bleaching was set with the 405-nm, 488-nm, and 555-nm laser lines at 100% output. Bleaching was started after 3 frames, and another 97 frames were taken every 320 ms during the recovery period. Values of mean ROI intensities were extracted with Zen Black software, exported to Microsoft Excel, and graphed with GraphPad Prism. To induce genuine stress granules in HuH-7 cells, the plasmid pEGFP-G3BP (kindly provided by Richard Lloyd, Baylor University), encoding an EGFP-G3BP1 fusion protein (85), was transfected with Lipofectamine 2000 reagent (Thermo Fischer Scientific) in cells plated in a 35-mm glass (no. 1.5)-bottom dish with a 27-mm viewing area (Nunc). After 24 h, cells containing G3BP1 granules were identified by live-cell microscopy on an LSM700 confocal system set to 37°C.

### Isolation of SNAP-nsP3 from cell lysates.

Stable CHIKV cells grown in six-well plates were labeled with BG-TMR-Star according to the live-cell staining protocol outlined above. Cells were collected by scraping them into PBS using plastic cell scrapers, followed by centrifugation in 1.5-ml microcentrifuge tubes. Cell pellets were lysed with 300 μl ice-cold Glasgow lysis buffer [1% Triton X-100, 120 mM KCl, 30 mM NaCl, 5 mM MgCl_2_, 10% glycerol, and 10 mM piperazine-*N*,*N*′-bis(2-ethanesulfonic acid) (PIPES)-NaOH, pH 7.2] containing protease inhibitors. Lysates were vortexed for 30 s for four cycles and returned to ice between cycles. A final spin at 850 × *g* was included to remove the remaining cellular debris. The final supernatant was added to a two-well Ibidi plastic slide with an Ibitreat surface for optimal cell adhesion (Ibidi). After an overnight incubation at 4°C, 1 ml of 4% formaldehyde was added to each well for 1 h at room temperature. Wells were washed with PBS, and images were captured with an LSM880 system operated in Fast Airyscan mode. The same protocol was used to analyze lysates from HuH-7 cells infected with CHIKV^ZsGreen-P3^.

### Bioimage analysis.

Data sets from Fig. 5B and 6B and from Video S1 in the supplemental material were processed with NIS-Elements AR imaging software (Nikon). Wide-field images were deconvolved within the Elements software according to the Richardson-Lucy algorithm (set to 10 iterations). All other microscopy images and videos were processed on the Icy (http://icy.bioimageanalysis.org) platform (84). Contrast was optimized in individual images by dragging the adjustable bounds of the histogram viewer, which enhances the contrast in each channel without altering the data (84). Color maps (cyan, magenta, green, gray, yellow, fire, or jet) were applied with the lookup table manager to each channel in combination with the corresponding histogram bounds. The HK-Means and Active Contours plugins (A. Dufour, V. Meas-Yedid, A. Grassart, and J. C. Olivo-Marin, presented at the 19th International Conference on Pattern Recognition, 8 to 11 December 2008) were used for segmentation of nsP3-containing granules, and the ROI viewer provided information about the maximum Feret diameter of the granules. Tracking of nsP3-containing granules was done with the Manual Tracking (written by Alexandre Dufour) plugin, which allows clicking on the center of individual granules for every time point and integration with the software's Track Manager (written by Fabrice de Chaumont) to visualize each track. Motion statistics (total displacement and relative/net displacement) for each track and peak velocity detected within an individual track were extracted from the Track Manager plugin (written by Fabrice de Chaumont) by adding the Motion Profiler track processor (written by Alexandre Dufour).

## Supplementary Material

Supplemental material

## References

[1] Hoarau JJ, Jaffar Bandjee MC, Krejbich Trotot P, Das T, Li-Pat-Yuen G, Dassa B, Denizot M, Guichard E, Ribera A, Henni T, Tallet F, Moiton MP, Gauzere BA, Bruniquet S, Jaffar Bandjee Z, Morbidelli P, Martigny G, Jolivet M, Gay F, Grandadam M, Tolou H, Vieillard V, Debre P, Autran B, Gasque P 2010 Persistent chronic inflammation and infection by chikungunya arthritogenic alphavirus in spite of a robust host immune response. J Immunol 184:5914–5927. doi:10.4049/jimmunol.0900255. 20404278

[2] Schilte C, Staikowsky F, Couderc T, Madec Y, Carpentier F, Kassab S, Albert ML, Lecuit M, Michault A 2013 Chikungunya virus-associated long-term arthralgia: a 36-month prospective longitudinal study. PLoS Negl Trop Dis 7:e2137. doi:10.1371/journal.pntd.0002137. 23556021 PMC3605278

[3] Javelle E, Ribera A, Degasne I, Gauzere BA, Marimoutou C, Simon F 2015 Specific management of post-chikungunya rheumatic disorders: a retrospective study of 159 cases in Reunion Island from 2006-2012. PLoS Negl Trop Dis 9:e0003603. doi:10.1371/journal.pntd.0003603. 25760632 PMC4356515

[4] Rodriguez-Morales AJ, Cardona-Ospina JA, Villamil-Gomez W, Paniz-Mondolfi AE 2015 How many patients with post-chikungunya chronic inflammatory rheumatism can we expect in the new endemic areas of Latin America? Rheumatol Int 35:2091–2094. doi:10.1007/s00296-015-3302-5. 26045218

[5] Rodriguez-Morales AJ, Gil-Restrepo AF, Ramirez-Jaramillo V, Montoya-Arias CP, Acevedo-Mendoza WF, Bedoya-Arias JE, Chica-Quintero LA, Murillo-Garcia DR, Garcia-Robledo JE, Castrillon-Spitia JD, Londono JJ, Bedoya-Rendon HD, Cardenas-Perez Jde J, Cardona-Ospina JA, Lagos-Grisales GJ 2016 Post-chikungunya chronic inflammatory rheumatism: results from a retrospective follow-up study of 283 adult and child cases in La Virginia, Risaralda, Colombia. F1000Res 5:360. doi:10.12688/f1000research.8235.2. 27081477 PMC4813633

[6] Sourisseau M, Schilte C, Casartelli N, Trouillet C, Guivel-Benhassine F, Rudnicka D, Sol-Foulon N, Le Roux K, Prevost MC, Fsihi H, Frenkiel MP, Blanchet F, Afonso PV, Ceccaldi PE, Ozden S, Gessain A, Schuffenecker I, Verhasselt B, Zamborlini A, Saib A, Rey FA, Arenzana-Seisdedos F, Despres P, Michault A, Albert ML, Schwartz O 2007 Characterization of reemerging chikungunya virus. PLoS Pathog 3:e89. doi:10.1371/journal.ppat.0030089. 17604450 PMC1904475

[7] Krejbich-Trotot P, Denizot M, Hoarau JJ, Jaffar-Bandjee MC, Das T, Gasque P 2011 Chikungunya virus mobilizes the apoptotic machinery to invade host cell defenses. FASEB J 25:314–325. doi:10.1096/fj.10-164178. 20881210

[8] Dhanwani R, Khan M, Alam SI, Rao PV, Parida M 2011 Differential proteome analysis of chikungunya virus-infected new-born mice tissues reveal implication of stress, inflammatory and apoptotic pathways in disease pathogenesis. Proteomics 11:1936–1951. doi:10.1002/pmic.201000500. 21472854

[9] Ozden S, Huerre M, Riviere JP, Coffey LL, Afonso PV, Mouly V, de Monredon J, Roger JC, El Amrani M, Yvin JL, Jaffar MC, Frenkiel MP, Sourisseau M, Schwartz O, Butler-Browne G, Despres P, Gessain A, Ceccaldi PE 2007 Human muscle satellite cells as targets of chikungunya virus infection. PLoS One 2:e527. doi:10.1371/journal.pone.0000527. 17565380 PMC1885285

[10] Hawman DW, Stoermer KA, Montgomery SA, Pal P, Oko L, Diamond MS, Morrison TE 2013 Chronic joint disease caused by persistent chikungunya virus infection is controlled by the adaptive immune response. J Virol 87:13878–13888. doi:10.1128/JVI.02666-13. 24131709 PMC3838294

[11] Labadie K, Larcher T, Joubert C, Mannioui A, Delache B, Brochard P, Guigand L, Dubreil L, Lebon P, Verrier B, de Lamballerie X, Suhrbier A, Cherel Y, Le Grand R, Roques P 2010 Chikungunya disease in nonhuman primates involves long-term viral persistence in macrophages. J Clin Invest 120:894–906. doi:10.1172/JCI40104. 20179353 PMC2827953

[12] Messaoudi I, Vomaske J, Totonchy T, Kreklywich CN, Haberthur K, Springgay L, Brien JD, Diamond MS, Defilippis VR, Streblow DN 2013 Chikungunya virus infection results in higher and persistent viral replication in aged rhesus macaques due to defects in anti-viral immunity. PLoS Negl Trop Dis 7:e2343. doi:10.1371/journal.pntd.0002343. 23936572 PMC3723534

[13] Poo YS, Rudd PA, Gardner J, Wilson JA, Larcher T, Colle MA, Le TT, Nakaya HI, Warrilow D, Allcock R, Bielefeldt-Ohmann H, Schroder WA, Khromykh AA, Lopez JA, Suhrbier A 2014 Multiple immune factors are involved in controlling acute and chronic chikungunya virus infection. PLoS Negl Trop Dis 8:e3354. doi:10.1371/journal.pntd.0003354. 25474568 PMC4256279

[14] Strauss JH, Strauss EG 1994 The alphaviruses: gene expression, replication, and evolution. Microbiol Rev 58:491–562. 7968923 10.1128/mr.58.3.491-562.1994 PMC372977

[15] Fros JJ, Pijlman GP 2016 Alphavirus infection: host cell shut-off and inhibition of antiviral responses. Viruses 8:E166. doi:10.3390/v8060166. 27294951 PMC4926186

[16] Fros JJ, Domeradzka NE, Baggen J, Geertsema C, Flipse J, Vlak JM, Pijlman GP 2012 Chikungunya virus nsP3 blocks stress granule assembly by recruitment of G3BP into cytoplasmic foci. J Virol 86:10873–10879. doi:10.1128/JVI.01506-12. 22837213 PMC3457282

[17] Panas MD, Varjak M, Lulla A, Eng KE, Merits A, Karlsson Hedestam GB, McInerney GM 2012 Sequestration of G3BP coupled with efficient translation inhibits stress granules in Semliki Forest virus infection. Mol Biol Cell 23:4701–4712. doi:10.1091/mbc.e12-08-0619. 23087212 PMC3521679

[18] Eckei L, Krieg S, Butepage M, Lehmann A, Gross A, Lippok B, Grimm AR, Kummerer BM, Rossetti G, Luscher B, Verheugd P 2017 The conserved macrodomains of the non-structural proteins of chikungunya virus and other pathogenic positive strand RNA viruses function as mono-ADP-ribosylhydrolases. Sci Rep 7:41746. doi:10.1038/srep41746. 28150709 PMC5288732

[19] McPherson RL, Abraham R, Sreekumar E, Ong SE, Cheng SJ, Baxter VK, Kistemaker HA, Filippov DV, Griffin DE, Leung AK 2017 ADP-ribosylhydrolase activity of chikungunya virus macrodomain is critical for virus replication and virulence. Proc Natl Acad Sci U S A 114:1666–1671. doi:10.1073/pnas.1621485114. 28143925 PMC5321000

[20] Mathur K, Anand A, Dubey SK, Sanan-Mishra N, Bhatnagar RK, Sunil S 2016 Analysis of chikungunya virus proteins reveals that non-structural proteins nsP2 and nsP3 exhibit RNA interference (RNAi) suppressor activity. Sci Rep 6:38065. doi:10.1038/srep38065. 27901124 PMC5128919

[21] Cristea IM, Carroll JW, Rout MP, Rice CM, Chait BT, MacDonald MR 2006 Tracking and elucidating alphavirus-host protein interactions. J Biol Chem 281:30269–30278. doi:10.1074/jbc.M603980200. 16895903

[22] Frolova E, Gorchakov R, Garmashova N, Atasheva S, Vergara LA, Frolov I 2006 Formation of nsP3-specific protein complexes during Sindbis virus replication. J Virol 80:4122–4134. doi:10.1128/JVI.80.8.4122-4134.2006. 16571828 PMC1440443

[23] Neuvonen M, Kazlauskas A, Martikainen M, Hinkkanen A, Ahola T, Saksela K 2011 SH3 domain-mediated recruitment of host cell amphiphysins by alphavirus nsP3 promotes viral RNA replication. PLoS Pathog 7:e1002383. doi:10.1371/journal.ppat.1002383. 22114558 PMC3219718

[24] Kim DY, Reynaud JM, Rasalouskaya A, Akhrymuk I, Mobley JA, Frolov I, Frolova EI 2016 New World and Old World alphaviruses have evolved to exploit different components of stress granules, FXR and G3BP proteins, for assembly of viral replication complexes. PLoS Pathog 12:e1005810. doi:10.1371/journal.ppat.1005810. 27509095 PMC4980055

[25] Scholte FE, Tas A, Albulescu IC, Zusinaite E, Merits A, Snijder EJ, van Hemert MJ 2015 Stress granule components G3BP1 and G3BP2 play a proviral role early in chikungunya virus replication. J Virol 89:4457–4469. doi:10.1128/JVI.03612-14. 25653451 PMC4442398

[26] Remenyi R, Roberts GC, Zothner C, Merits A, Harris M 2017 SNAP-tagged chikungunya virus replicons improve visualisation of non-structural protein 3 by fluorescence microscopy. Sci Rep 7:5682. doi:10.1038/s41598-017-05820-0. 28720784 PMC5515888

[27] Roberts GC, Zothner C, Remenyi R, Merits A, Stonehouse NJ, Harris M 2017 Evaluation of a range of mammalian and mosquito cell lines for use in chikungunya virus research. Sci Rep 7:14641. doi:10.1038/s41598-017-15269-w. 29116243 PMC5677012

[28] Utt A, Das PK, Varjak M, Lulla V, Lulla A, Merits A 2015 Mutations conferring a noncytotoxic phenotype on chikungunya virus replicons compromise enzymatic properties of nonstructural protein 2. J Virol 89:3145–3162. doi:10.1128/JVI.03213-14. 25552719 PMC4337533

[29] Remenyi R, Gao Y, Hughes RE, Curd A, Zothner C, Peckham M, Merits A, Harris M 2018 Persistent chikungunya virus replication in human cells is associated with presence of stable cytoplasmic granules containing non-structural protein 3. bioRxiv doi:10.1101/236703. PMC6069192 29875241

[30] Jain S, Wheeler JR, Walters RW, Agrawal A, Barsic A, Parker R 2016 ATPase-modulated stress granules contain a diverse proteome and substructure. Cell 164:487–498. doi:10.1016/j.cell.2015.12.038. 26777405 PMC4733397

[31] Wheeler JR, Matheny T, Jain S, Abrisch R, Parker R 2016 Distinct stages in stress granule assembly and disassembly. Elife 5:e18413. doi:10.7554/eLife.18413. 27602576 PMC5014549

[32] Muller CB, Enderlein J 2010 Image scanning microscopy. Phys Rev Lett 104:198101. doi:10.1103/PhysRevLett.104.198101. 20867000

[33] Sheppard CJ, Mehta SB, Heintzmann R 2013 Superresolution by image scanning microscopy using pixel reassignment. Opt Lett 38:2889–2892. doi:10.1364/OL.38.002889. 23903171

[34] Huff J 2015 The Airyscan detector from ZEISS: confocal imaging with improved signal-to-noise ratio and super-resolution. Nat Methods 12:1205. doi:10.1038/nmeth.f.388.

[35] Schulte T, Liu L, Panas MD, Thaa B, Dickson N, Gotte B, Achour A, McInerney GM 2016 Combined structural, biochemical and cellular evidence demonstrates that both FGDF motifs in alphavirus nsP3 are required for efficient replication. Open Biol 6:160078. doi:10.1098/rsob.160078. 27383630 PMC4967826

[36] Frolova EI, Gorchakov R, Pereboeva L, Atasheva S, Frolov I 2010 Functional Sindbis virus replicative complexes are formed at the plasma membrane. J Virol 84:11679–11695. doi:10.1128/JVI.01441-10. 20826696 PMC2977861

[37] Vognsen T, Moller IR, Kristensen O 2013 Crystal structures of the human G3BP1 NTF2-like domain visualize FxFG Nup repeat specificity. PLoS One 8:e80947. doi:10.1371/journal.pone.0080947. 24324649 PMC3852005

[38] Panas MD, Schulte T, Thaa B, Sandalova T, Kedersha N, Achour A, McInerney GM 2015 Viral and cellular proteins containing FGDF motifs bind G3BP to block stress granule formation. PLoS Pathog 11:e1004659. doi:10.1371/journal.ppat.1004659. 25658430 PMC4450067

[39] York AG, Chandris P, Nogare DD, Head J, Wawrzusin P, Fischer RS, Chitnis A, Shroff H 2013 Instant super-resolution imaging in live cells and embryos via analog image processing. Nat Methods 10:1122–1126. doi:10.1038/nmeth.2687. 24097271 PMC3898876

[40] McInerney GM, Kedersha NL, Kaufman RJ, Anderson P, Liljestrom P 2005 Importance of eIF2alpha phosphorylation and stress granule assembly in alphavirus translation regulation. Mol Biol Cell 16:3753–3763. doi:10.1091/mbc.e05-02-0124. 15930128 PMC1182313

[41] Agapov EV, Frolov I, Lindenbach BD, Pragai BM, Schlesinger S, Rice CM 1998 Noncytopathic Sindbis virus RNA vectors for heterologous gene expression. Proc Natl Acad Sci U S A 95:12989–12994. 9789028 10.1073/pnas.95.22.12989 PMC23682

[42] Casales E, Rodriguez-Madoz JR, Ruiz-Guillen M, Razquin N, Cuevas Y, Prieto J, Smerdou C 2008 Development of a new noncytopathic Semliki Forest virus vector providing high expression levels and stability. Virology 376:242–251. doi:10.1016/j.virol.2008.03.016. 18442838

[43] Pohjala L, Utt A, Varjak M, Lulla A, Merits A, Ahola T, Tammela P 2011 Inhibitors of alphavirus entry and replication identified with a stable chikungunya replicon cell line and virus-based assays. PLoS One 6:e28923. doi:10.1371/journal.pone.0028923. 22205980 PMC3242765

[44] Varjak M, Zusinaite E, Merits A 2010 Novel functions of the alphavirus nonstructural protein nsP3 C-terminal region. J Virol 84:2352–2364. doi:10.1128/JVI.01540-09. 20015978 PMC2820926

[45] Frolov I, Kim DY, Akhrymuk M, Mobley JA, Frolova EI 2017 Hypervariable domain of Eastern equine encephalitis virus nsP3 redundantly utilizes multiple cellular proteins for replication complex assembly. J Virol 91:e00371-17. doi:10.1128/JVI.00371-17. 28468889 PMC5487569

[46] Fros JJ, Geertsema C, Zouache K, Baggen J, Domeradzka N, van Leeuwen DM, Flipse J, Vlak JM, Failloux AB, Pijlman GP 2015 Mosquito Rasputin interacts with chikungunya virus nsP3 and determines the infection rate in Aedes albopictus. Parasit Vectors 8:464. doi:10.1186/s13071-015-1070-4. 26384002 PMC4573678

[47] Gorchakov R, Garmashova N, Frolova E, Frolov I 2008 Different types of nsP3-containing protein complexes in Sindbis virus-infected cells. J Virol 82:10088–10101. doi:10.1128/JVI.01011-08. 18684830 PMC2566286

[48] Peranen J, Laakkonen P, Hyvonen M, Kaariainen L 1995 The alphavirus replicase protein nsP1 is membrane-associated and has affinity to endocytic organelles. Virology 208:610–620. doi:10.1006/viro.1995.1192. 7747433

[49] Salonen A, Vasiljeva L, Merits A, Magden J, Jokitalo E, Kaariainen L 2003 Properly folded nonstructural polyprotein directs the Semliki Forest virus replication complex to the endosomal compartment. J Virol 77:1691–1702. doi:10.1128/JVI.77.3.1691-1702.2003. 12525603 PMC140886

[50] Ahola T, Lampio A, Auvinen P, Kaariainen L 1999 Semliki Forest virus mRNA capping enzyme requires association with anionic membrane phospholipids for activity. EMBO J 18:3164–3172. doi:10.1093/emboj/18.11.3164. 10357827 PMC1171397

[51] Spuul P, Balistreri G, Kaariainen L, Ahola T 2010 Phosphatidylinositol 3-kinase-, actin-, and microtubule-dependent transport of Semliki Forest virus replication complexes from the plasma membrane to modified lysosomes. J Virol 84:7543–7557. doi:10.1128/JVI.00477-10. 20484502 PMC2897599

[52] Grimley PM, Berezesky IK, Friedman RM 1968 Cytoplasmic structures associated with an arbovirus infection: loci of viral ribonucleic acid synthesis. J Virol 2:1326–1338. 5750316 10.1128/jvi.2.11.1326-1338.1968 PMC375472

[53] Friedman RM, Levin JG, Berezesky IK, Grimley PM 1972 Membrane-associated replication complex in arbovirus infection. J Virol 10:504–515. 4342056 10.1128/jvi.10.3.504-515.1972 PMC356492

[54] Froshauer S, Kartenbeck J, Helenius A 1988 Alphavirus RNA replicase is located on the cytoplasmic surface of endosomes and lysosomes. J Cell Biol 107:2075–2086. doi:10.1083/jcb.107.6.2075. 2904446 PMC2115628

[55] Sawicki DL, Sawicki SG 1980 Short-lived minus-strand polymerase for Semliki Forest virus. J Virol 34:108–118. 6768898 10.1128/jvi.34.1.108-118.1980 PMC288676

[56] Sawicki SG, Sawicki DL, Kaariainen L, Keranen S 1981 A Sindbis virus mutant temperature-sensitive in the regulation of minus-strand RNA synthesis. Virology 115:161–172. doi:10.1016/0042-6822(81)90098-2. 7292988

[57] Gotte B, Liu L, McInerney GM 2018 The enigmatic alphavirus non-structural protein 3 (nsP3) revealing its secrets at last. Viruses 10:E105. doi:10.3390/v10030105. 29495654 PMC5869498

[58] Hellstrom K, Vihinen H, Kallio K, Jokitalo E, Ahola T 2015 Correlative light and electron microscopy enables viral replication studies at the ultrastructural level. Methods 90:49–56. doi:10.1016/j.ymeth.2015.04.019. 25916619

[59] Liss V, Barlag B, Nietschke M, Hensel M 2015 Self-labelling enzymes as universal tags for fluorescence microscopy, super-resolution microscopy and electron microscopy. Sci Rep 5:17740. doi:10.1038/srep17740. 26643905 PMC4672345

[60] Fros JJ, Liu WJ, Prow NA, Geertsema C, Ligtenberg M, Vanlandingham DL, Schnettler E, Vlak JM, Suhrbier A, Khromykh AA, Pijlman GP 2010 Chikungunya virus nonstructural protein 2 inhibits type I/II interferon-stimulated JAK-STAT signaling. J Virol 84:10877–10887. doi:10.1128/JVI.00949-10. 20686047 PMC2950581

[61] Akhrymuk I, Kulemzin SV, Frolova EI 2012 Evasion of the innate immune response: the Old World alphavirus nsP2 protein induces rapid degradation of Rpb1, a catalytic subunit of RNA polymerase II. J Virol 86:7180–7191. doi:10.1128/JVI.00541-12. 22514352 PMC3416352

[62] Chang CW, Lee CP, Su MT, Tsai CH, Chen MR 2015 BGLF4 kinase modulates the structure and transport preference of the nuclear pore complex to facilitate nuclear import of Epstein-Barr virus lytic proteins. J Virol 89:1703–1718. doi:10.1128/JVI.02880-14. 25410863 PMC4300756

[63] Gong D, Kim YH, Xiao Y, Du Y, Xie Y, Lee KK, Feng J, Farhat N, Zhao D, Shu S, Dai X, Chanda SK, Rana TM, Krogan NJ, Sun R, Wu TT 2016 A herpesvirus protein selectively inhibits cellular mRNA nuclear export. Cell Host Microbe 20:642–653. doi:10.1016/j.chom.2016.10.004. 27832591 PMC5130111

[64] Varjak M, Saul S, Arike L, Lulla A, Peil L, Merits A 2013 Magnetic fractionation and proteomic dissection of cellular organelles occupied by the late replication complexes of Semliki Forest virus. J Virol 87:10295–10312. doi:10.1128/JVI.01105-13. 23864636 PMC3754020

[65] Lussignol M, Kopp M, Molloy K, Vizcay-Barrena G, Fleck RA, Dorner M, Bell KL, Chait BT, Rice CM, Catanese MT 2016 Proteomics of HCV virions reveals an essential role for the nucleoporin Nup98 in virus morphogenesis. Proc Natl Acad Sci U S A 113:2484–2489. doi:10.1073/pnas.1518934113. 26884193 PMC4780614

[66] Neufeldt CJ, Joyce MA, Levin A, Steenbergen RH, Pang D, Shields J, Tyrrell DL, Wozniak RW 2013 Hepatitis C virus-induced cytoplasmic organelles use the nuclear transport machinery to establish an environment conducive to virus replication. PLoS Pathog 9:e1003744. doi:10.1371/journal.ppat.1003744. 24204278 PMC3814334

[67] Reineke LC, Dougherty JD, Pierre P, Lloyd RE 2012 Large G3BP-induced granules trigger eIF2alpha phosphorylation. Mol Biol Cell 23:3499–3510. doi:10.1091/mbc.e12-05-0385. 22833567 PMC3442399

[68] Kedersha N, Stoecklin G, Ayodele M, Yacono P, Lykke-Andersen J, Fritzler MJ, Scheuner D, Kaufman RJ, Golan DE, Anderson P 2005 Stress granules and processing bodies are dynamically linked sites of mRNP remodeling. J Cell Biol 169:871–884. doi:10.1083/jcb.200502088. 15967811 PMC2171635

[69] Kedersha N, Cho MR, Li W, Yacono PW, Chen S, Gilks N, Golan DE, Anderson P 2000 Dynamic shuttling of TIA-1 accompanies the recruitment of mRNA to mammalian stress granules. J Cell Biol 151:1257–1268. doi:10.1083/jcb.151.6.1257. 11121440 PMC2190599

[70] Gretton SN, Taylor AI, McLauchlan J 2005 Mobility of the hepatitis C virus NS4B protein on the endoplasmic reticulum membrane and membrane-associated foci. J Gen Virol 86:1415–1421. doi:10.1099/vir.0.80768-0. 15831953

[71] Jones DM, Gretton SN, McLauchlan J, Targett-Adams P 2007 Mobility analysis of an NS5A-GFP fusion protein in cells actively replicating hepatitis C virus subgenomic RNA. J Gen Virol 88:470–475. doi:10.1099/vir.0.82363-0. 17251564

[72] Wolk B, Buchele B, Moradpour D, Rice CM 2008 A dynamic view of hepatitis C virus replication complexes. J Virol 82:10519–10531. doi:10.1128/JVI.00640-08. 18715913 PMC2573176

[73] Panas MD, Ivanov P, Anderson P 2016 Mechanistic insights into mammalian stress granule dynamics. J Cell Biol 215:313–323. doi:10.1083/jcb.201609081. 27821493 PMC5100297

[74] Molliex A, Temirov J, Lee J, Coughlin M, Kanagaraj AP, Kim HJ, Mittag T, Taylor JP 2015 Phase separation by low complexity domains promotes stress granule assembly and drives pathological fibrillization. Cell 163:123–133. doi:10.1016/j.cell.2015.09.015. 26406374 PMC5149108

[75] Alberti S, Mateju D, Mediani L, Carra S 2017 Granulostasis: protein quality control of RNP granules. Front Mol Neurosci 10:84. doi:10.3389/fnmol.2017.00084. 28396624 PMC5367262

[76] Tsetsarkin K, Higgs S, McGee CE, De Lamballerie X, Charrel RN, Vanlandingham DL 2006 Infectious clones of chikungunya virus (La Reunion isolate) for vector competence studies. Vector Borne Zoonotic Dis 6:325–337. doi:10.1089/vbz.2006.6.325. 17187566

[77] Bourett TM, Sweigard JA, Czymmek KJ, Carroll A, Howard RJ 2002 Reef coral fluorescent proteins for visualizing fungal pathogens. Fungal Genet Biol 37:211–220. doi:10.1016/S1087-1845(02)00524-8. 12431456

[78] Nakabayashi H, Taketa K, Miyano K, Yamane T, Sato J 1982 Growth of human hepatoma cells lines with differentiated functions in chemically defined medium. Cancer Res 42:3858–3863. 6286115

[79] Schonborn J, Oberstrass J, Breyel E, Tittgen J, Schumacher J, Lukacs N 1991 Monoclonal antibodies to double-stranded RNA as probes of RNA structure in crude nucleic acid extracts. Nucleic Acids Res 19:2993–3000. doi:10.1093/nar/19.11.2993. 2057357 PMC328262

[80] Bodor DL, Rodriguez MG, Moreno N, Jansen LE 2012 Analysis of protein turnover by quantitative SNAP-based pulse-chase imaging. Curr Protoc Cell Biol Chapter 8:Unit8.8. doi:10.1002/0471143030.cb0808s55. 23129118

[81] Curd A, Cleasby A, Makowska K, York A, Shroff H, Peckham M 2015 Construction of an instant structured illumination microscope. Methods 88:37–47. doi:10.1016/j.ymeth.2015.07.012. 26210400 PMC4641873

[82] Munch B, Trtik P, Marone F, Stampanoni M 2009 Stripe and ring artifact removal with combined wavelet–Fourier filtering. Opt Express 17:8567–8591. doi:10.1364/OE.17.008567. 19434191

[83] Sage D, Donati L, Soulez F, Fortun D, Schmit G, Seitz A, Guiet R, Vonesch C, Unser M 2017 DeconvolutionLab2: an open-source software for deconvolution microscopy. Methods 115:28–41. doi:10.1016/j.ymeth.2016.12.015. 28057586

[84] de Chaumont F, Dallongeville S, Chenouard N, Herve N, Pop S, Provoost T, Meas-Yedid V, Pankajakshan P, Lecomte T, Le Montagner Y, Lagache T, Dufour A, Olivo-Marin JC 2012 Icy: an open bioimage informatics platform for extended reproducible research. Nat Methods 9:690–696. doi:10.1038/nmeth.2075. 22743774

[85] White JP, Cardenas AM, Marissen WE, Lloyd RE 2007 Inhibition of cytoplasmic mRNA stress granule formation by a viral proteinase. Cell Host Microbe 2:295–305. doi:10.1016/j.chom.2007.08.006. 18005751

