# Supplementary material for: Persistent Replication of a Chikungunya Virus Replicon in Human Cells Is Associated with Presence of Stable Cytoplasmic Granules Containing Nonstructural Protein 3
Source: J Virol. 2018 Jul 31;92(16):e00477-18. doi: 10.1128/JVI.00477-18 (PMC6069192; doi:10.1128/JVI.00477-18)
Supplement: Supplemental material [file supp_92_16_e00477-18__index.html]

Persistent Replication of a Chikungunya Virus Replicon in Human Cells Is Associated with Presence of Stable Cytoplasmic Granules Containing Nonstructural Protein 3 — Supplemental material 

# Persistent Replication of a Chikungunya Virus Replicon in Human Cells Is Associated with Presence of Stable Cytoplasmic Granules Containing Nonstructural Protein 3

## Supplemental material

- Supplemental file 1 -

  Video S1 (Time-lapse video of SNAP-nsP3 in stable CHIKV cells by wide-field microscopy.)

  MP4, 15M
- Supplemental file 2 -

  Video S2 (Time-lapse video of SNAP-nsP3 in a stable CHIKV cell by instant structured illumination microscopy [iSIM].)

  MP4, 19M
- Supplemental file 3 -

  Video S3 (Time-lapse video of SNAP-nsP3 in stable CHIKV cells by iSIM [tracks 1 to 3].)

  MP4, 17M
- Supplemental file 4 -

  Video S4 (Time-lapse video of SNAP-nsP3 in stable CHIKV cells by iSIM [tracks 4 to 7].)

  MP4, 19M
- Supplemental file 5 -

  Video S5 (Time-lapse video of SNAP-nsP3 in stable CHIKV cells by iSIM [tracks 8 to 10].)

  MP4, 19M
- Supplemental file 6 -

  Video S6 (Time-lapse video of SNAP-nsP3 in stable CHIKV cells by total internal reflection fluorescence [TIRF] microscopy.)

  MP4, 19M
- Supplemental file 7 -

  Supplemental Video Legends.

  PDF, 186K
