# Supplementary material for: Persistent Replication of a Chikungunya Virus Replicon in Human Cells Is Associated with Presence of Stable Cytoplasmic Granules Containing Nonstructural Protein 3
Source: J Virol. 2018 Jul 31;92(16):e00477-18. doi: 10.1128/JVI.00477-18 (PMC6069192; doi:10.1128/JVI.00477-18)
Supplement: Supplemental material [file JVI.00477-18_zjv016183763s7.pdf]

Supplemental Material – Legends for videos

Video S1. Time-lapse of SNAP-nsP3 in stable CHIKV cells by widefield microscopy. Time-lapse movie of ZsGreen (green) and BG-SiR staining of SNAP-nsP3 (orange). Individual frames from these live-cell recordings are shown in Fig. 5B. This is followed by a time-lapse movie of the SNAP-nsP3 channel (gray) with the ZsGreen channel digitally hidden. Z-stacks were taken every 30 min for 16 h, alternating in the green and far-red channel. Widefield images were deconvolved within the Nikon Elements software according to the Richardson-Lucy algorithm.

Video S2. Time-lapse of SNAP-nsP3 in a stable CHIKV cell by instant structured illumination microscopy (iSIM). Video shows a two-color snapshot of a ZsGreen-positive cell stained for SNAP-nsP3 (orange), followed by a time-lapse recording of SNAP-nsP3 (grayscale). Zoomed-in views of moving nsP3-granule is shown as well (with a colored track either overlaid or hidden).

Video S3. Time-lapse of SNAP-nsP3 in stable CHIKV cells by instant structured illumination microscopy (iSIM). Zoomed-in views showing Tracks 1-3. Views in which tracks were hidden are provided as well (“Track X hidden”). Note that these cropped time-lapse recordings underwent deconvolution as outlined in ‘Materials and Methods’ section, whereas Video S2 shows images that were not processed for deconvolution.

Video S4. Time-lapse of SNAP-nsP3 in stable CHIKV cells by instant structured illumination microscopy (iSIM). Zoomed-in views showing Tracks 4-7.

Video S5. Time-lapse of SNAP-nsP3 in stable CHIKV cells by instant structured illumination microscopy (iSIM). Zoomed-in views showing Tracks 8-10.

Video S6. Time-lapse of SNAP-nsP3 in stable CHIKV cells by total internal reflection fluorescence (TIRF) microscopy. Plasma membrane was stained with CellMask Orange (magenta). Individual frames from these live-cell recordings are shown in Fig. 6B.
